# Silica nanoparticle-loaded thermoresponsive block copolymer vesicles: a new post-polymerization encapsulation strategy and thermally triggered release†

**DOI:** 10.1039/d2sc02103j

**Published:** 2022-08-08

**Authors:** Adam Czajka, Sarah J. Byard, Steven P. Armes

**Affiliations:** Dainton Building, The University of Sheffield Brook Hill Sheffield S3 7HF UK s.p.armes@sheffield.ac.uk

## Abstract

A thermoresponsive amphiphilic diblock copolymer that can form spheres, worms or vesicles in aqueous media at neutral pH by simply raising the dispersion temperature from 1 °C (spheres) to 25 °C (worms) to 50 °C (vesicles) is prepared *via* polymerization-induced self-assembly (PISA). Heating such an aqueous copolymer dispersion from 1 °C up to 50 °C in the presence of 19 nm glycerol-functionalized silica nanoparticles enables this remarkable ‘shape-shifting’ behavior to be exploited as a new post-polymerization encapsulation strategy. The silica-loaded vesicles formed at 50 °C are then crosslinked using a disulfide-based dihydrazide reagent. Such covalent stabilization enables the dispersion to be cooled to room temperature without loss of the vesicle morphology, thus aiding characterization and enabling the loading efficiency to be determined as a function of both copolymer and silica concentration. Small-angle X-ray scattering (SAXS) analysis indicated a mean vesicle membrane thickness of approximately 20 ± 2 nm for the linear vesicles and TEM studies confirmed encapsulation of the silica nanoparticles within these nano-objects. After removal of the non-encapsulated silica nanoparticles *via* multiple centrifugation–redispersion cycles, thermogravimetric analysis indicated that vesicle loading efficiencies of up to 86% can be achieved under optimized conditions. Thermally-triggered release of the silica nanoparticles is achieved by cleaving the disulfide bonds at 50 °C using tris(2-carboxyethyl)phosphine (TCEP), followed by cooling to 20 °C to induce vesicle dissociation. SAXS is also used to confirm the release of silica nanoparticles by monitoring the disappearance of the structure factor peak arising from silica–silica interactions.

## Abstract 2

A loading efficiency of up to 86% is achieved for silica nanoparticles encapsulated within crosslinkable redox-sensitive thermoresponsive diblock copolymer vesicles in water at 50 °C; triggered release is also demonstrated for this system.

## Graphical abstract

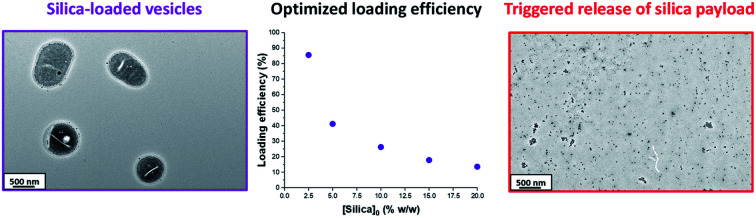

## Introduction

The pioneering studies of Discher and Eisenberg^1–3^ has led to significant interest in block copolymer vesicles for many potential applications, including microencapsulation.^4–11^ However, such nano-objects are typically prepared in relatively dilute solution *via* post-polymerization processing.^12^ Over the past decade or so, polymerization-induced self-assembly (PISA) has become widely recognized as a powerful platform technology for the rational design of functional block copolymer nano-objects of controlled size and morphology.^13–30^ In the case of aqueous formulations, PISA involves growing a hydrophobic polymer from one end of a water-soluble polymer precursor to produce an amphiphilic diblock copolymer. As the second block grows, it becomes insoluble at some critical chain length, which induces *in situ* self-assembly to produce sterically-stabilized diblock copolymer nanoparticles. In principle, spheres, worms or vesicles can be formed, with the final copolymer morphology typically depending on various synthesis parameters such as the mean DP of each block, the copolymer concentration and the chemical nature of each block.^31–36^ Such amphiphilic diblock copolymers can be conveniently prepared using reversible addition–fragmentation chain transfer (RAFT) polymerization.^37–43^

It is well-known that PISA syntheses based on RAFT aqueous emulsion polymerization often lead to kinetically-trapped spheres.^15,34,44,45^ There are various strategies that enable this morphological constraint to be overcome^46–50^ but the resulting worms or vesicles normally do not exhibit thermoresponsive character. On the other hand, PISA syntheses based on RAFT aqueous dispersion polymerization necessarily involve using less hydrophobic (water-miscible) vinyl monomers.^28^ This typically leads to the formation of thermoresponsive diblock copolymer nano-objects whose preferred morphology can be adjusted by systematic variation of the solution temperature. For example, a 10% aqueous dispersion of poly(glycerol monomethacrylate)-poly(2-hydroxypropyl methacrylate) [PGMA-PHPMA] worms forms a soft gel at 20 °C that undergoes degelation on cooling to 5 °C owing to a worm-to-sphere transition.^13^ This change in copolymer morphology is reversible and can be rationalized in terms of surface plasticization of the worm cores by water, which leads to a subtle reduction in the fractional packing parameter for the copolymer chains.^51,52^

By targeting a longer PHPMA block, Blanazs *et al.* were able to prepare polydisperse PGMA-PHPMA vesicles.^53^ Moreover, TEM studies indicated that a transient ‘jellyfish-like’ structure was involved in the morphological evolution from spheres to worms to vesicles that occurs during such PISA syntheses.^53^ In view of this unexpected observation, we conjectured that if PGMA-PHPMA vesicles were prepared in the presence of silica nanoparticles, some of these nanoparticles would diffuse within these ‘jellyfish-like’ structures and be retained during vesicle formation. This hypothesis proved to be correct, with the *in situ* encapsulation of both silica nanoparticles and various globular proteins/enzymes being subsequently reported by several research groups.^21,24,54^ Moreover, thermally-triggered release of the silica nanoparticle payload has been demonstrated in the case of PGMA-PHPMA vesicles, which undergo a vesicle-to-sphere transition on cooling below ambient temperature.^54,55^ However, the proportion of silica nanoparticles encapsulated within the vesicles relative to the initial silica concentration was relatively low at 8–11%.^54^ This parameter is designated as the loading efficiency, *LE*_TGA_ (as determined by thermogravimetry analysis, or TGA). For such calculations, it is assumed that (i) all the copolymer chains self-assemble to form vesicles, (ii) there are no empty vesicles, and (iii) all the excess (non-encapsulated) silica nanoparticles are removed *via* centrifugation.^54^

**Fig. 1:**
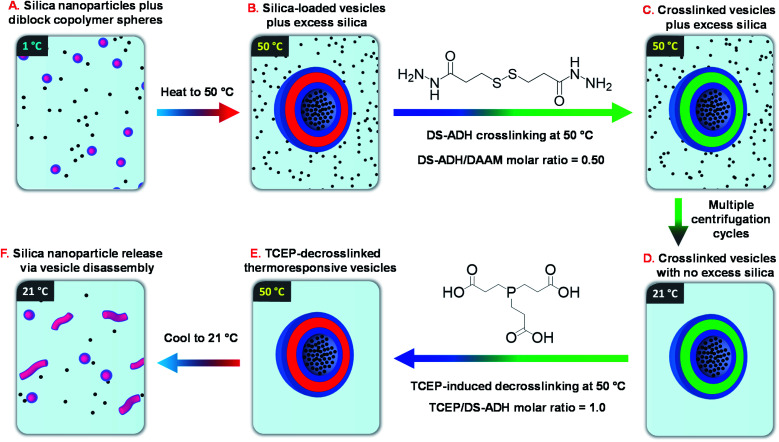
Schematic overview of the six stages involved in the encapsulation and release of silica nanoparticles using a thermoresponsive ‘shape-shifting’ PDMAC_56_-P(HBA-*stat*-DAAM)_254_ diblock copolymer.

**Scheme 1:**
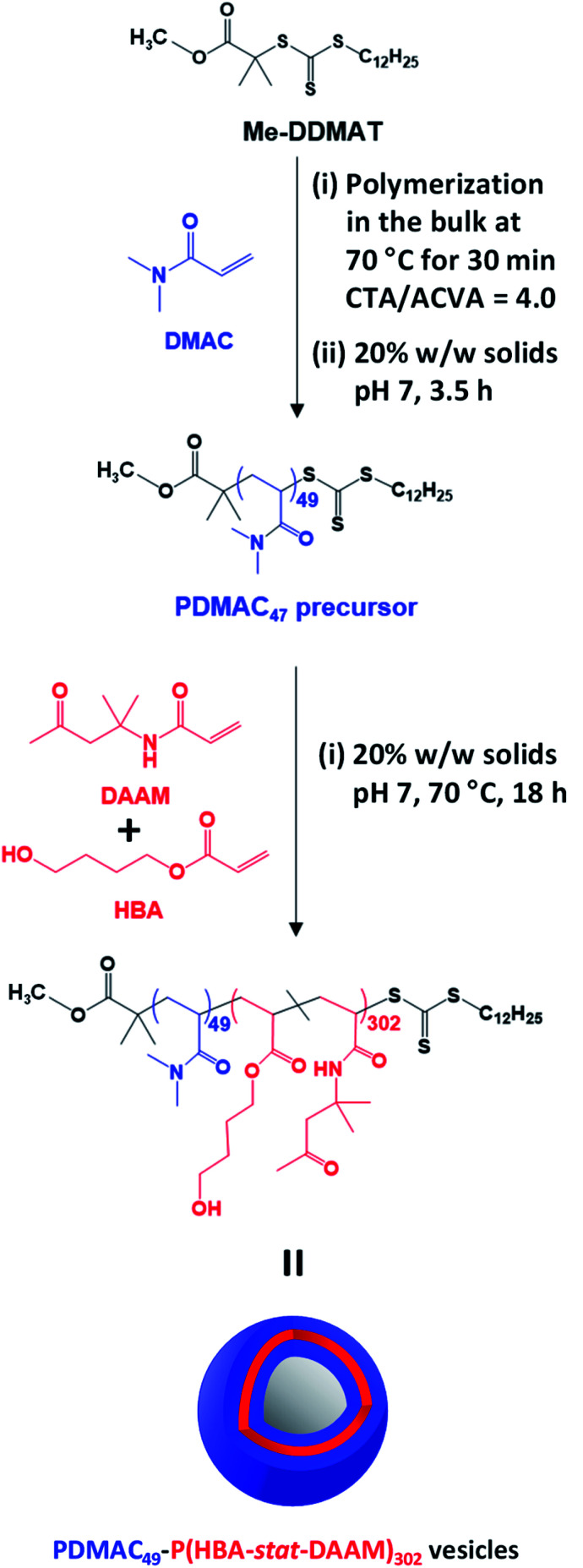
Reaction scheme for the synthesis of the PDMAC_49_ precursor *via* RAFT aqueous solution polymerization of DMAC at 70 °C using a Me-DDMAT RAFT agent and an ACVA initiator. This PDMAC_49_ precursor was subsequently chain-extended *via* RAFT aqueous dispersion copolymerization of a binary mixture of 80 mol% HBA and 20 mol% DAAM to produce PDMAC_49_-P(HBA-*stat*-DAAM)_302_ vesicles at pH 7.

Recently, we reported the remarkable aqueous self-assembly behavior exhibited by a *single* amphiphilic diblock copolymer of fixed composition.^14^ More specifically, simply varying the solution temperature enabled a poly(*N*,*N*-dimethylacrylamide)_56_-(4-hydroxybutyl acrylate-*stat*-diacetone acrylamide)_254_ [PDMAC_56_-P(HBA-*stat*-DAAM)_254_] diblock copolymer to form spheres (1 °C), worms (25 °C), vesicles (50 °C) or lamellae (70 °C) with excellent thermoreversibility even at 0.1% w/w copolymer concentration. One drawback for this new system was the requirement to crosslink such nano-objects at the desired temperature using a water-soluble reagent (adipic acid dihydrazide, ADH). Such covalent stabilization was essential for characterization of the vesicle morphology by transmission electron microscopy (TEM). Moreover, it also enabled an aqueous dispersion of vesicles to be cooled to ambient temperature without reverting to worms or spheres. In view of this unprecedented self-assembly behavior, we hypothesized that such ‘shape-shifting’ thermoresponsive amphiphilic diblock copolymers might offer an opportunity to develop a much more efficient post-polymerization vesicle loading protocol rather than *in situ* loading during PISA. More specifically, the freeze-dried copolymer is added to an aqueous dispersion of glycerol-functionalized silica nanoparticles to produce self-assembled spherical copolymer nanoparticles at sub-ambient temperature (see Fig. 1A). Subsequent heating induces a sphere-to-vesicle transition that should result in the *in situ* encapsulation of at least some of the silica nanoparticles as a model payload (see Fig. 1B). This concept is explored in the present study, for which two critically important refinements are introduced. Firstly, the original carboxylic acid-based RAFT agent is replaced with a methyl ester analog(see Scheme 1), which ensures that the desired thermoresponsive behavior occurs at pH 7, rather than at pH 3. This subtle change in chemical structure is essential if such vesicles act to serve as suitable nanocontainers for enzymes or other bioactive (macro)molecules.^11,12^ Secondly, we introduce a disulfide-based crosslinker^51^ that enables the covalent stabilization of these vesicles to be reversed by adding a suitable reagent (TCEP) to cleave the disulfide bonds within the crosslinks (see Fig. 1C–E). In principle, this should provide a new payload release mechanism for such redox-sensitive vesicles, as summarized in Fig. 1F.

## Results and discussion

### Synthesis and characterization of PDMAC-P(HBA-*stat*-DAAM) vesicles

Methanol was used to esterify DDMAT in dichloromethane to produce the methyl ester analog, Me-DDMAT, as reported previously.^56^ The crude product was purified by column chromatography and the mean degree of esterification was determined to be 98% by comparing the integrated intensities of signals *a* and *b* , which were assigned to the terminal methyl group on the dodecyl chain and the methyl ester protons, respectively (see Fig. S1†). As reported by Byard and co-workers,^56^ this modification of the RAFT agent eliminates the pH sensitivity conferred by ionization of the terminal carboxylic acid group located on the steric stabilizer chains, which would otherwise lead to the formation of anionic spheres at neutral pH.^57^

Accordingly, Me-DDMAT was used to prepare thermoresponsive PDMAC_49_-P(HBA-*stat*-DAAM)_302_ diblock copolymer nano-objects at pH 7 *via* a highly convenient and efficient one-pot protocol (see Scheme 1 and ESI† for further details). Unfortunately, Me-DDMAT is not soluble in water so the DMAC polymerization was initially conducted in the bulk to enable the monomer to act as a co-solvent for this RAFT agent. After 30 min at 70 °C, degassed water was added to convert this bulk polymerization into a RAFT aqueous solution polymerization, while simultaneously reducing the viscosity of the reaction mixture. After a further 3.5 h, a small aliquot of the resulting water-soluble PDMAC homopolymer was removed for analysis. ^1^H NMR spectroscopy studies confirmed that more than 99% DMAC conversion was achieved, see Fig. S2.† The mean DP of this PDMAC precursor was determined to be 49 *via* end-group analysis by UV spectroscopy using the linear calibration plot shown in Fig. S3.†

Once the PDMAC_49_ precursor had been obtained, the second step involved the RAFT aqueous dispersion copolymerization of 80 mol% HBA with 20 mol% DAAM at 70 °C to produce PDMAC_49_-P(HBA-*stat*-DAAM)_302_ vesicles in the form of a 20% w/w aqueous dispersion at pH 7, see Scheme 1. ^1^H NMR studies indicated that more than 99% conversion was achieved for both comonomers and this technique was also used to calculate the mean DP for the membrane-forming P(HBA-*stat*-DAAM)_302_ block *via* end-group analysis (see Fig. S4†). DMF GPC analysis confirmed efficient chain extension for the PDMAC_56_ precursor and a relatively narrow molecular weight distribution (*M*_w_/*M*_n_ = 1.24) for the final diblock copolymer (see Fig. S5†).

**Fig. 2:**
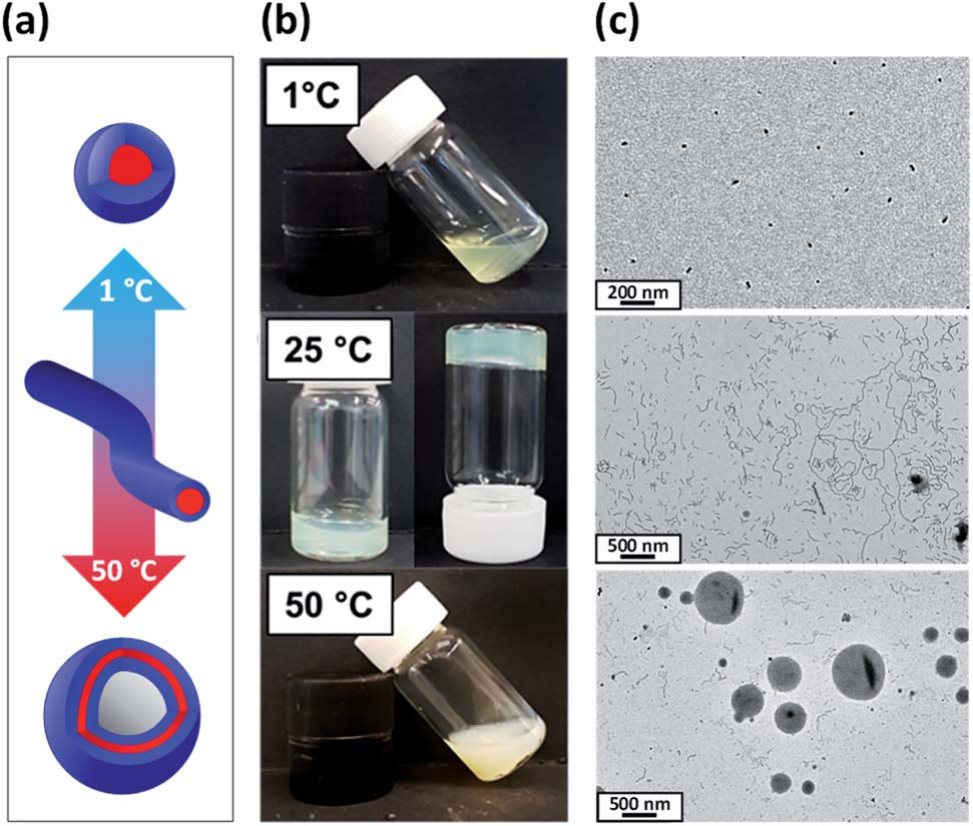
(a) Schematic representation of the reversible morphological transitions exhibited by a 20% w/w aqueous dispersion of ‘shape-shifting’ PDMAC_49_-P(HBA-*stat*-DAAM)_302_ nano-objects on varying the temperature from 1 °C to 50 °C. (b) Digital photographs illustrating the change in physical appearance of a 20% w/w aqueous dispersion of PDMAC_49_-P(HBA-*stat*-DAAM)_302_ nano-objects recorded at 1 °C, 25 °C and 50 °C (after allowing 30 min for thermal equilibrium at each temperature). (c) Corresponding TEM images recorded for 0.10% w/w aqueous dispersions of the same PDMAC_49_-P(HBA-*stat*-DAAM)_302_ nano-objects after their covalent stabilization at the desired temperature using a disulfide-based adipic acid dihydrazide [DS-ADH] crosslinker at a DS-ADH/DAAM molar ratio of 0.50 (spheres crosslinked at 1 °C, worms crosslinked at 25 °C, and vesicles crosslinked at 50 °C).

The themoresponsive nature of these PDMAC_49_-P(HBA-*stat*-DAAM)_302_ nano-objects is readily apparent by inspection of the visual appearance of a 20% w/w aqueous dispersion at 1 °C, 25 °C and 50 °C, see Fig. 2b. A transparent free-flowing dispersion is obtained on cooling to 1 °C, indicating the presence of non-interacting spheres. In contrast, a semi-transparent free-standing gel is formed at 25 °C owing to a 3D network of weakly interacting worms.^58^ Finally, a turbid free-flowing dispersion is observed on heating to 50 °C, which is consistent with the formation of non-interacting vesicles.

**Scheme 2:**
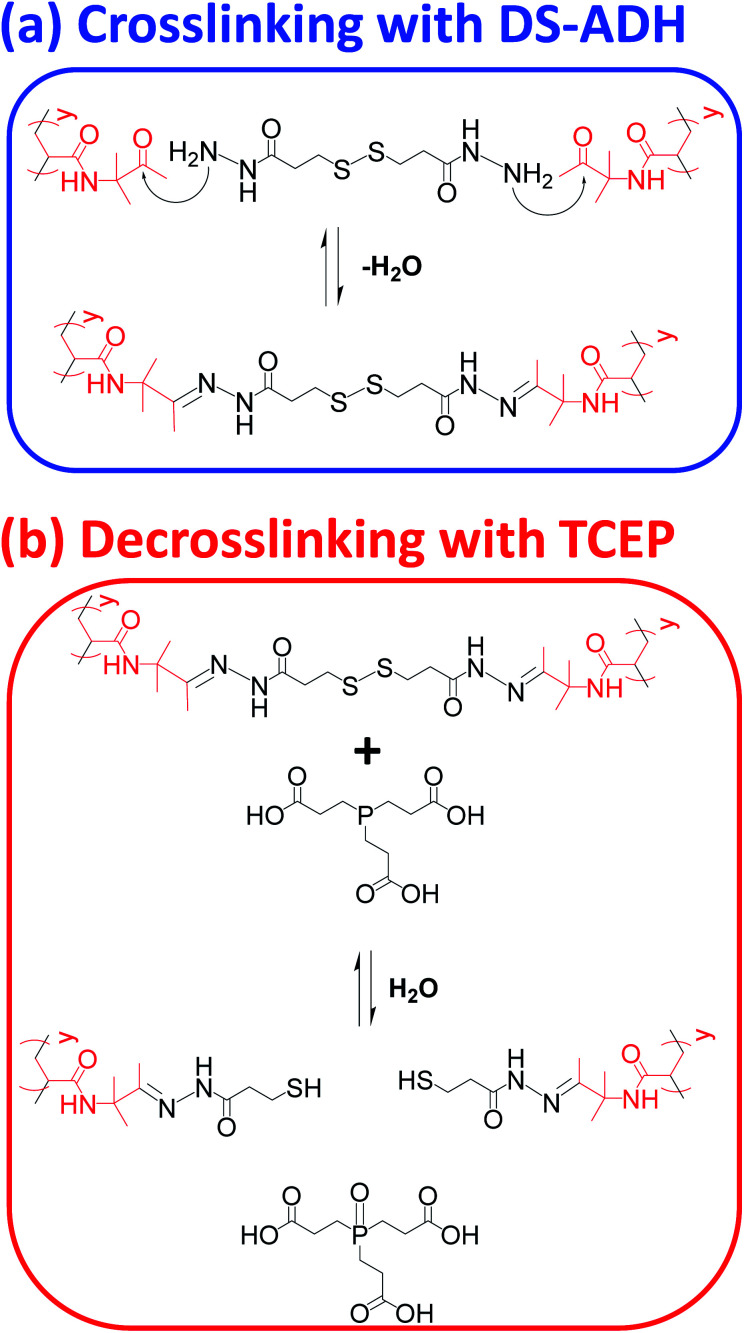
(a) Nucleophilic attack of pendent ketone groups on DAAM repeat units by DS-ADH, which leads to crosslinks between the hydrophobic P(HBA-*stat*-DAAM)_302_ chains. (b) Reductive cleavage of disulfide bonds within these crosslinks using tris(2-carboxyethyl)phosphine (TCEP).

In principle, TEM studies should reveal the various morphologies formed by this amphiphilic PDMAC_49_-P(HBA-*stat*-DAAM)_302_ diblock copolymer. Unfortunately, the relatively low glass transition temperature of the core-forming block leads to film formation on the TEM grid.^14^ To circumvent this problem, the nano-objects are covalently stabilized by reacting the pendent ketone groups on the DAAM repeat units with 3,3′-dithiobis(propanoicdihydrazide) (DS-ADH) at pH 7, see Scheme 2a. Such crosslinking eliminates the thermoresponsive behavior exhibited by this diblock copolymer, thus permanently fixing whichever copolymer morphology is predominant at the crosslinking temperature.^59^ More specifically, this protocol enabled visualization of pure spheres, worms or vesicles by TEM when crosslinking was conducted at 1, 25 or 50 °C, respectively (see Fig. 2c). Variable temperature dynamic light scattering (DLS) studies performed on a 0.10% w/w aqueous dispersion prior to crosslinking indicated *z*-average diameters of 52 nm (PDI = 0.31), 119 nm (PDI = 0.21) and 214 nm (PDI = 0.25) at 1, 25 and 50 °C respectively, see Fig. S6.† However, only a ‘sphere-equivalent’ diameter is reported in the case of the worms, with this parameter corresponding to neither the worm contour length nor the worm cross-sectional thickness.^60^ Aqueous dispersions prepared at 1% w/w solids were then covalently stabilized using DS-ADH at 1, 25 or 50 °C and DLS studies were performed at 25 °C after ten-fold dilution to produce 0.10% w/w dispersions, see Fig. S6.† Essentially the same z-average diameters are observed compared to those obtained in the variable temperature DLS experiments performed on the corresponding linear thermoresponsive nano-objects, indicating that successful covalent stabilization is achieved when using the DS-ADH crosslinker. Small-angle X-ray scattering (SAXS) was used to analyse a 1.0% w/w aqueous dispersion of PDMAC_49_-P(HBA-*stat*-DAAM)_302_ vesicles at 50 °C, see Fig. S7.† A satisfactory fit to the scattering pattern was obtained using a well-known vesicle scattering model (see ESI†).^61^ An overall volume-average diameter of 202 nm was calculated, which is in good agreement with a z-average vesicle diameter of 214 nm reported by DLS studies conducted at 50 °C. Furthermore, SAXS analysis suggests a mean vesicle membrane thickness of 20 ± 2 nm, which is comparable to the mean worm diameter estimated by TEM (Fig. 2c).

**Fig. 3:**
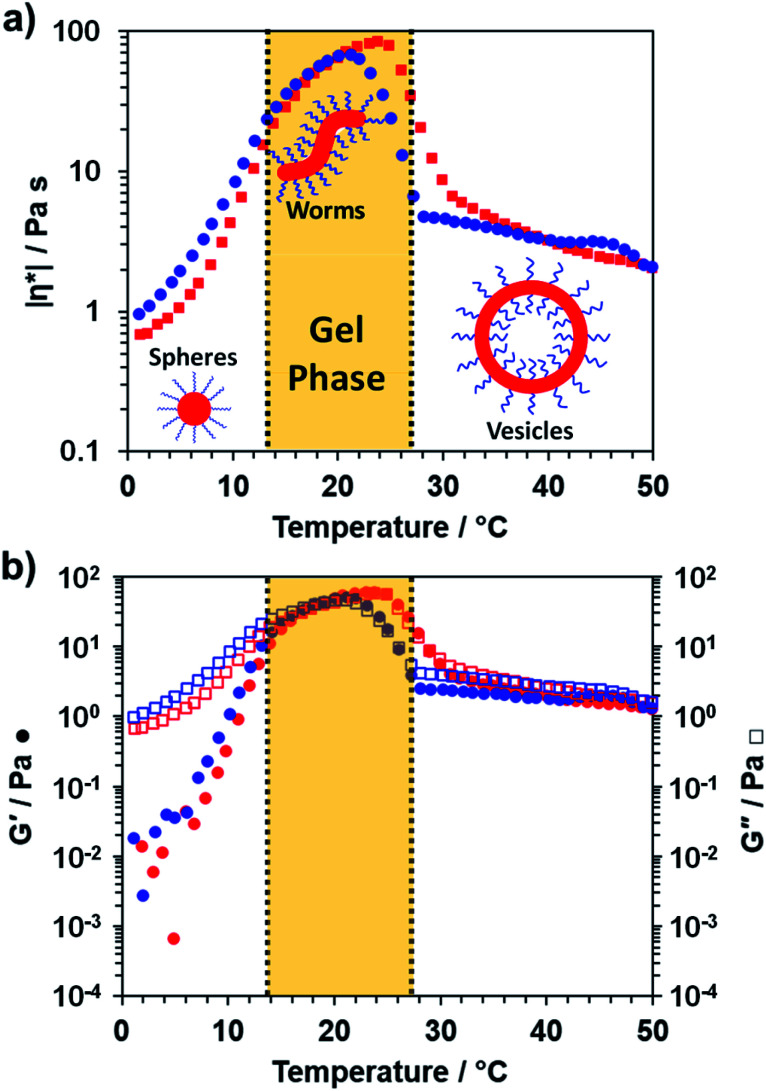
Variable temperature rheological data recorded for a 20% w/w aqueous dispersion of PDMAC_49_-P(HBA-*stat*-DAAM)_302_ nano-objects at an applied strain of 1.0% and an angular frequency of 1.0 rad s^−1^. This aqueous dispersion was equilibrated at 1 °C for 15 min prior to a 1 °C to 50 °C to 1 °C thermal cycle at 1 °C min^−1^: red and blue data points correspond to the heating and cooling runs respectively, while vertical dashed lines represent approximate phase boundaries for the three morphologies. (a) Variation in complex viscosity, |η*|, with temperature and (b) variation in *G*′ and *G*′′ with temperature.

To further characterize the thermoresponsive behavior exhibited by this PDMAC_49_-P(HBA-*stat*-DAAM)_302_ diblock copolymer, rheology studies were conducted on a 20% w/w aqueous dispersion as a function of temperature (see Fig. 3). A low-viscosity fluid was obtained at 1 °C, which is consistent with the presence of the free-flowing spheres indicated by TEM studies (see Fig. 2c). Warming the dispersion induced a sol–gel transition at 18 °C owing to the formation of worms. Further heating to 29 °C led to *in situ* degelation and a significant reduction in dispersion viscosity as a result of a worm-to-vesicle transition. The thermoresponsive nature of this diblock copolymer was examined by variable temperature DLS studies conducted in the presence and absence of 20% w/w silica nanoparticles. In such experiments, vesicles were formed at 50 °C, then converted into spheres at 1 °C before reforming the vesicles at 50 °C. DLS particle size distributions (data not shown) obtained for 0.10% w/w dispersions at each of these three stages indicated little or no difference in the z-average diameter obtained for the original empty vesicles and the reconstituted vesicles. Moreover, very similar results were obtained in the presence of 20% w/w silica nanoparticles, which indicates that the latter do not adversely affect the *in situ* block copolymer self-assembly.

### Encapsulation of silica nanoparticles

A series of initial copolymer and silica concentrations were evaluated for the encapsulation of silica nanoparticles within PDMAC_49_-P(HBA-*stat*-DAAM)_302_ vesicles, see Table 1. First, this amphiphilic diblock copolymer was cooled to 1 °C to induce a vesicle-to-sphere transition. The resulting spheres were then mixed with an aqueous dispersion of glycerol-functionalized silica nanoparticles (Bindzil CC401, 19 nm diameter) that had been previously cooled to the same temperature (Fig. 1A). After remaining at 1 °C for 2 h, the resulting binary mixture of diblock copolymer spheres and silica nanoparticles was heated to 50 °C to induce vesicle formation (Fig. 1B). After equilibrating for 2 h, the vesicles were then crosslinked using DS-ADH at 50 °C for 24 h (Fig. 1C). After crosslinking, DLS intensity distributions shows the presence of two populations corresponding to excess silica nanoparticles and crosslinked vesicles, see Fig. S8.† This DS-ADH crosslinker was prepared according to a literature protocol^62^ (see ESI† for further details and Fig. S9† for the corresponding ^1^H NMR spectra). In the present work, redox-active DS-ADH is preferred to ADH^59^ because its central disulfide bond can be cleaved using tris(2-carboxyethyl)phosphine) [TCEP]. In principle, such decrosslinking should restore the thermoresponsive character of the diblock copolymer chains and thus enable release of the encapsulated silica nanoparticles to be achieved on cooling owing to a vesicle-to-worm or vesicle-to-sphere transition. To investigate the kinetics of DS-ADH crosslinking, a 5.0% w/w aqueous dispersion of empty PDMAC_49_-P(HBA-*stat*-DAAM)_302_ vesicles were crosslinked at 50 °C and a DAAM/DS-ADH molar ratio of 2.0. Aliquots were removed at various time intervals and immediately diluted to 0.1% w/v in methanol, which is a good solvent for both PDMAC and PDAAM. Thus, once crosslinking has occurred, molecular dissolution can no longer occur and particles should be observed by DLS. The DLS derived count rate is related to the particle diameter and a plateau is observed after approximately 60 min, suggesting that crosslinking is complete (see Fig. S10†).

**Table 1:** Summary of DLS *z*-average particle diameters, *D*_*z*_ (after DS-ADH crosslinking and removal of excess silica nanoparticles), mean silica contents determined by thermogravimetry (TGA) and TGA-derived silica loading efficiency (*LE*_TGA_) for a series of PDMAC_49_-P(HBA-*stat*-DAAM)_302_ vesicles prepared at copolymer concentrations of 5–25% w/w in the presence of various concentrations of silica nanoparticles

[Copolymer]_0_ (% w/w)	[Silica]_0_ (% w/w)	*D*_*z*_ (nm)	TGA silica content (%)	*LE*_TGA_ (%)
10	2.5	1045 (0.28)	16.9	85.6
10	5	1509 (0.07)	16.4	41.1
10	10	2631 (0.39)	19.9	26.2
10	15	2592 (0.31)	20.2	17.8
10	20	1586 (0.29)	20.5	13.5
5	20	1066 (0.11)	20.3	13.4
10	20	1432 (0.13)	21.8	14.7
15	20	1235 (0.11)	22.7	15.4
20	20	1874 (0.08)	24.3	16.9
25	20	1515 (0.13)	24.6	17.2

**Fig. 4:**
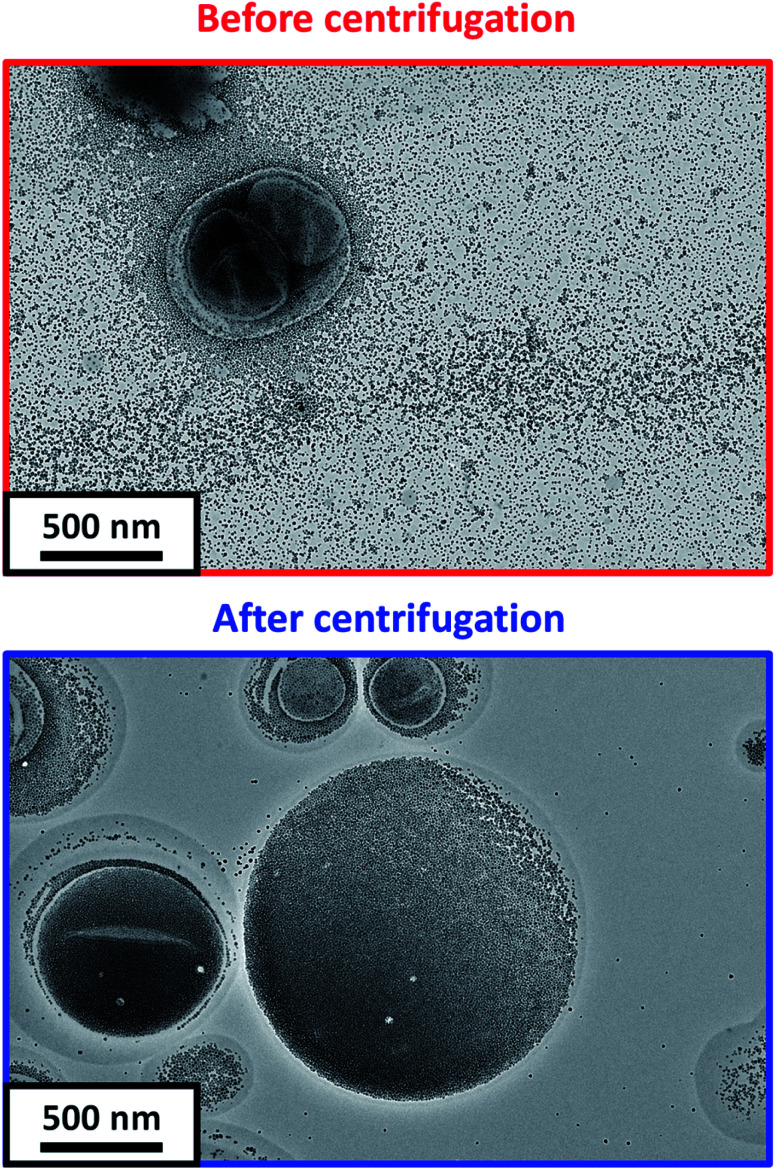
Representative TEM images recorded for DS-ADH crosslinked PDMAC_49_-P(HBA-*stat*-DAAM)_302_ vesicles prepared at a copolymer concentration of 5.0% w/w in the presence of a 20% w/w aqueous dispersion of silica nanoparticles (a) before and (b) after ten centrifugation–redispersion cycles to remove excess silica nanoparticles.

After DS-ADH crosslinking of the silica-loaded PDMAC_49_-P(HBA-*stat*-DAAM)_302_ vesicles, excess non-encapsulated silica nanoparticles were removed by diluting the PDMAC_49_-P(HBA-*stat*-DAAM)_302_ dispersions to 1.0% w/w and centrifuging at 9000 rpm for 5 min (Fig. 1D). The aqueous supernatant was carefully removed and the sedimented vesicles were redispersed in deionized water at pH 7. To investigate the minimum number of centrifugation–redispersion cycles required to remove the excess silica nanoparticles, TEM images were recorded for each supernatant solution. Little or no free silica was detected after ten cycles, see Fig. S11a.† Moreover, the derived count rate (or scattered light intensity) determined by DLS became essentially constant within eight cycles, see Fig. S11b.† After ten cycles, TEM analysis shows clear evidence of silica encapsulation within the (unstained) vesicles with minimal excess free silica (see Fig. 4). One reviewer of this manuscript commented on the significant size difference observed for vesicles formed in the presence and absence of the silica nanoparticles. However, this size difference is not necessarily attributable to the silica nanoparticles. For example, Warren *et al.* reported that similar thermoresponsive diblock copolymer vesicles were relatively large and polydisperse when prepared *via* an aqueous PISA formulation. However, much smaller, less polydisperse vesicles were formed after performing a single thermal cycle (involving a vesicle-to-sphere-vesicle transition) in the absence of any silica nanoparticles.^63^ Moreover, it is also likely that the vesicle dimensions depend on additional parameters such as the copolymer concentration.

**Fig. 5:**
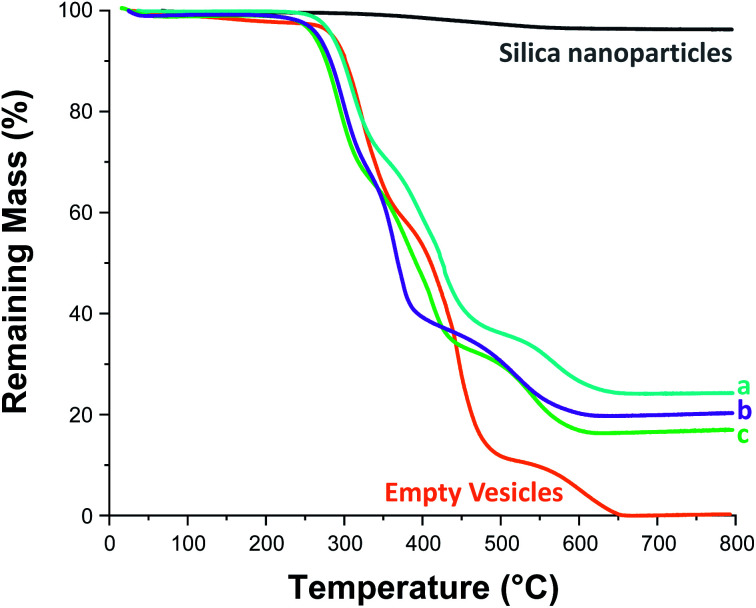
Thermogravimetric analysis curves recorded for Bindzil CC401 glycerol-functionalized 19 nm silica nanoparticles (black line), dried PDMAC_49_-P(HBA-*stat*-DAAM)_302_ vesicles (orange line), and three examples of silica-loaded crosslinked PDMAC_49_-P(HBA-*stat*-DAAM)_302_ vesicles after purification *via* ten centrifugation–redispersion cycles: (a) [copolymer]_0_ = 20% w/w, [silica]_0_ = 20% w/w; (b) [copolymer]_0_ = 10% w/w, [silica]_0_ = 10% w/w; (c) [copolymer]_0_ = 10% w/w, [silica]_0_ = 2.5% w/w.

On removal of the excess silica nanoparticles, a small aliquot of each purified vesicle dispersion (see Table 1) was retained for thermogravimetric analysis (TGA), see Fig. S12 and S13.† First, each aliquot was freeze-dried overnight. The dried silica-loaded PDMAC_49_-P(HBA-*stat*-DAAM)_302_ vesicles were then heated up to 800 °C in air to pyrolyse the organic component, leaving only the thermally stablesilica nanoparticles as a residue (see Fig. 5). In a control experiment, empty PDMAC_49_-P(HBA-*stat*-DAAM)_302_ vesicles were also heated to 800 °C in air. No residual mass was detected in this case, confirming that copolymer pyrolysis is complete under such conditions (see Fig. 5). Thus the vesicle loading efficiency, *LE*_TGA_, can be calculated from the mass of residual silica determined by TGA, see Table 1. However, these particular silica nanoparticles lose 3.8% mass on heating up to 800 °C in air (see Fig. 5). This is attributed to (i) loss of surface moisture and (ii) pyrolysis of the surface glycerol groups on the silica nanoparticles. This mass loss must be taken into account when calculating the loading efficiency for the silica-loaded vesicles (see Section 2.1 in the ESI† for a model calculation).

**Fig. 6:**
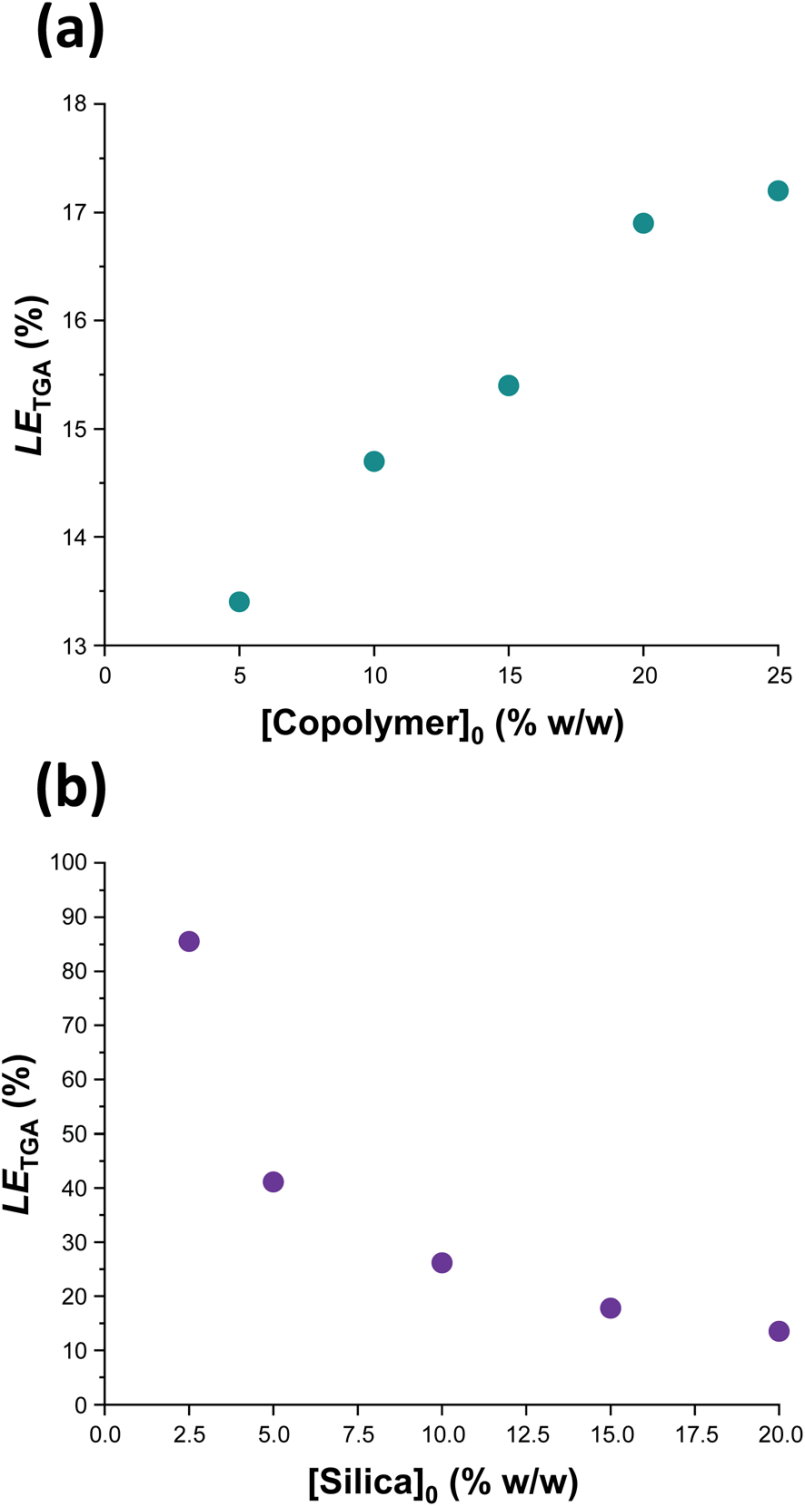
Vesicle loading efficiency, *LE*_TGA_, as determined by thermogravimetric analysis for: (a) a series of silica-loaded PDMAC_49_-P(HBA-*stat*-DAAM)_302_ vesicles prepared at various copolymer concentrations using a constant silica nanoparticle concentration of 20% w/w; (b) a series of PDMAC_49_-P(HBA-*stat*-DAAM)_302_ vesicles prepared at various silica nanoparticle concentrations using a constant copolymer concentration of 10% w/w. See Section 2.1 of the ESI† for further details of the *LE*_TGA_ calculation.

Previously, Mable *et al.* studied the encapsulation of the same 19 nm glycerol-functionalized silica nanoparticles within PGMA_58_-PHPMA_250_ vesicles prepared *via* RAFT aqueous dispersion polymerization of HPMA. However, the highest *LE*_TGA_ obtained for this prior *in situ* approach was only 10.5% (and the mean value was around 9%).^54^ Mable *et al.* chose to use a constant copolymer concentration of 10% w/w while varying the silica nanoparticle concentration from zero to 35% w/w. Similarly, we chose to vary the silica nanoparticle concentration while employing a fixed copolymer concentration of 10% w/w, see Table 1 and Fig. 6b. Importantly, lowering the silica nanoparticle concentration from 20% w/w to 2.5% w/w led to a substantial improvement in *LE*_TGA_ from 13.5% to 86%. The latter value is significantly higher than that obtained by Mable *et al.*,^54^ which suggests that the new post-polymerization encapsulation strategy reported herein is significantly more efficient than *in situ* encapsulation during PISA. For the sample with the highest encapsulation efficiency (86%), we calculate the concentration of silica nanoparticles encapsulated within the vesicles to be 1.94% w/w, which is slightly lower than the initial silica concentration (2.5% w/w). This suggests that there is no enrichment of the silica nanoparticles during their encapsulation. Furthermore, we calculate that, on average, there are 1356 silica nanoparticles within each vesicle. To examine the proposed encapsulation strategy for bioactive species, both silica encapsulation and crosslinking were conducted at 37 °C on two separate aqueous dispersions containing an initial copolymer concentration of 10% w/w, and initial silica concentrations of 5 and 2.5% w/w respectively, *i.e.* equivalent to rows 1 & 2 in Table 1. Silica encapsulation efficiencies of 63.6% and 27.0% were determined by thermogravimetric analysis for initial silica concentrations of 2.5 and 5% w/w respectively. This is lower than encapsulation efficiencies reported at 50 °C for the same formulations (85.6% and 41.1% respectively). However, DLS studies indicate that smaller vesicles are obtained when crosslinking is conducted at 37 °C. For example, at initial copolymer and silica concentrations of 10 and 5% w/w respectively, particle diameters are 1509 nm and 1324 nm at 50 °C and 37 °C, respectively. This reduction in vesicle size is consistent with the lower encapsulation efficiency observed at 37 °C. Nevertheless, the encapsulation protocol can be performed under relatively mild conditions, which should enable bioactive species such as enzymes to be loaded within such redox-sensitive vesicles while minimizing denaturation. In this context, it is worth emphasizing that enzyme encapsulation is typically conducted at relatively low enzyme concentrations,^21^ which corresponds to the conditions at which this new vesicle loading protocol appears to be most efficient.

In a second set of vesicle encapsulation experiments, the silica nanoparticle concentration was held constant at 20% w/w while the copolymer concentration was systematically varied from 5 to 25% w/w, see Table 1 and Fig. 6b. In this case, *LE*_TGA_ increased monotonically from 13.4 to 17.2%. One reason for this relatively modest increase may be that higher copolymer concentrations are likely to favor the formation of oligolamellar vesicles (*i.e.* vesicles within vesicles), see Fig. S14.† If so, this may be detrimental to the concomitant encapsulation of silica nanoparticles. Moreover, it is noteworthy that entries 5 and 7 in Table 1 correspond to identical formulations: both experiments were performed using a copolymer concentration of 10% w/w and a silica nanoparticle concentration of 20% w/w. Hence the corresponding *LE*_TGA_ values of 14.7% and 13.5% indicate reasonably good reproducibility for the vesicle loading efficiency (and the efficiency of removal of excess silica nanoparticles). In this case, the minor discrepancy is presumably related to the modest difference in mean vesicle diameter (616 nm *vs.* 708 nm, see Table 1) indicated by DLS studies, with the larger vesicles again facilitating a higher loading efficiency. Further experiments were undertaken to assess the reproducibility of the loading efficiency data under selected conditions (see Table S1†). Satisfactory agreement (±5%) was observed in most cases.

### Silica nanoparticle release

To monitor the release of the silica nanoparticle payload, a purified 1.0% w/w aqueous dispersion of DS-ADH crosslinked, silica-loaded PDMAC_49_-P(HBA-*stat*-DAAM)_302_ vesicles was heated up to 50 °C followed by the addition of a stoichiometric amount of TCEP (TCEP/DS-ADH molar ratio = 1.0, Fig. 1E). TCEP cleaves the disulfide bonds within the crosslinks, which restores the thermoresponsive character observed for the original linear vesicles, see Scheme 2b. Thus, cooling the TCEP-treated vesicles to below 20 °C induces a vesicle-to-worm or vesicle-to-sphere transition and hence releases the encapsulated silica nanoparticles. This was confirmed by DLS studies conducted at 1 °C on 0.1% w/w aqueous dispersions of PDMAC_49_-P(HBA-*stat*-DAAM)_302_ nano-objects before and after TCEP addition, which indicated z-average particle diameters of 1066 nm and 64 nm respectively. Furthermore, TEM studies of the same TCEP-treated dried dispersion confirmed the presence of free silica nanoparticles but did not provide any evidence for the diblock copolymer spheres. This is precisely as expected: crosslinking is essential to image such diblock copolymer nano-objects by TEM whereas TCEP-induced decrosslinking simply leads to their film formation owing to the low-*T*_g_ copolymer chains.^14^ It is perhaps worth emphasizing that the silica nanoparticles remain encapsulated within the vesicles indefinitely in the absence of any TCEP. This is because they are too large to diffuse through the vesicle membrane. Furthermore, TEM analysis conducted on the same sample after storage for approximately six weeks at 20 °C show no evidence for silica release (data not shown).

In principle, SAXS should be an ideal technique for probing the change in copolymer morphology associated with the release of the silica nanoparticle payload. According to Mable and co-workers,^54^ the encapsulation of silica nanoparticles within the vesicles should produce a prominent structure factor owing to interactions between the spatially confined nanoparticles. After TCEP treatment with subsequent cooling to a suitably low temperature to induce either a vesicle-to-worm or vesicle-to-sphere transition (see Fig. 3), this structure factor should be gradually lost as the encapsulated silica nanoparticles are released into the aqueous continuous phase.

**Fig. 7:**
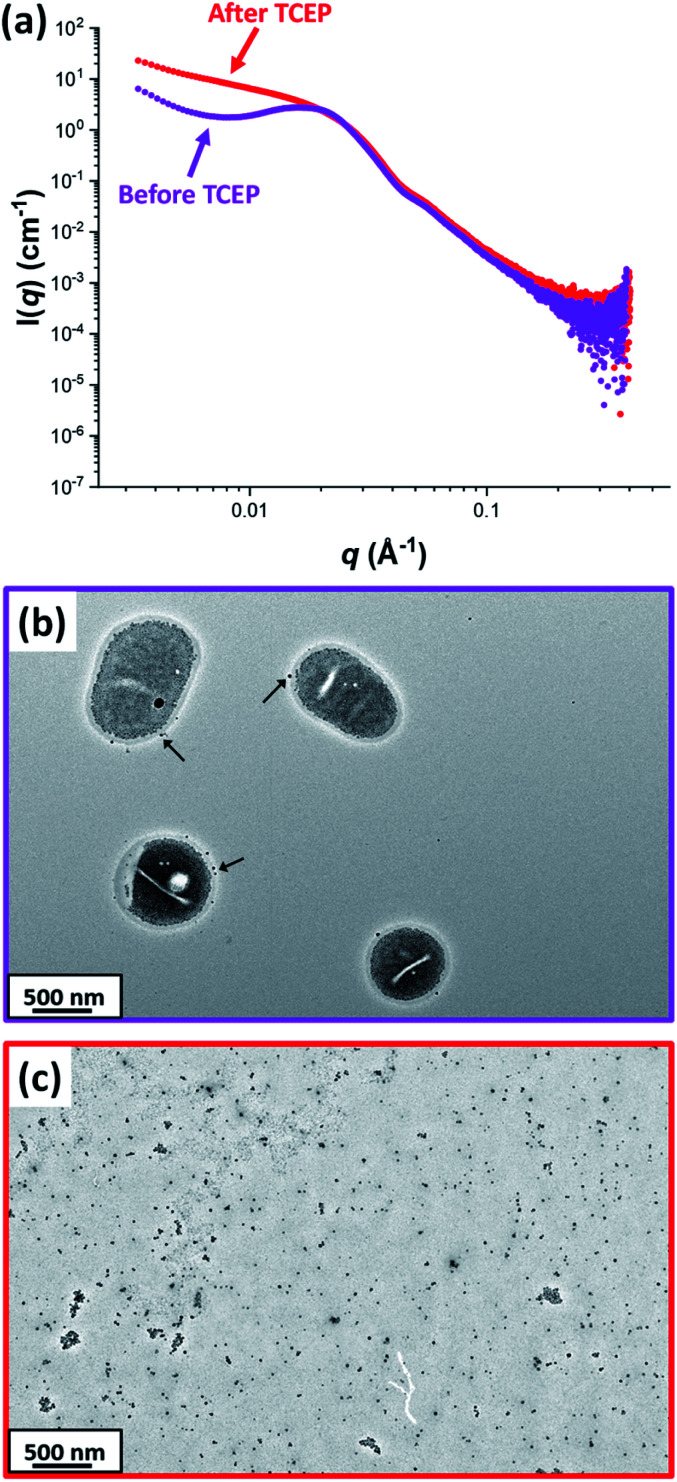
(a) SAXS patterns recorded for a 1.0% w/w aqueous dispersion of PDMAC_49_-P(HBA-*stat*-DAAM)_302_ vesicles (loaded with 2.5% w/w silica nanoparticles) before (purple pattern) and after TCEP addition (red pattern) confirming loss of the structure factor corresponding to the silica nanoparticles encapsulated within the vesicles. The low *q* gradient in the SAXS pattern recorded at 20 °C indicates the formation of worms after decrosslinking. Corresponding TEM images recorded (b) before and (c) after TCEP addition confirm the presence of silica-loaded vesicles and free silica nanoparticles, respectively. The black arrows shown in (b) indicate a few silica nanoparticles apparently adsorbed to the outer surface of the vesicles. [N.B. The linear worms formed at 20 °C in (c) cannot be observed by TEM because they undergo film formation during TEM grid preparation].

Fig. 7A shows the X-ray scattering intensity, I(*q*), plotted as a function of the scattering vector, *q* [where *q* = (4π sin *θ*)/*λ*, *λ* is the X-ray radiation wavelength and sin *θ* is half of the scattering angle], for SAXS patterns recorded at 20 °C before and after TCEP addition. A prominent structure factor is observed at *q* ∼0.02 Å^−1^ prior to TCEP addition, which corresponds to inter-particle interactions arising from the encapsulated silica nanoparticles.^54,55^ After TCEP addition, this structure factor disappears and a scattering pattern corresponding to PDMAC_49_-P(HBA-*stat*-DAAM)_302_ worms is obtained at 20 °C. This is consistent with the data shown in Fig. 2 and 3. Clearly, the disulfide bonds within the crosslinks are cleaved on exposure to a TCEP/DS-ADH molar ratio of 1.0, which restores the thermoresponsive character of the amphiphilic diblock copolymer chains. TEM analysis of the dried diluted copolymer dispersion after TCEP addition confirmed the presence of free silica nanoparticles (see Fig. 7C). However, the linear copolymer worms that are formed at 20 °C undergo *in situ* film formation on the TEM grid and hence cannot be imaged. The black arrows shown in Fig. 7b indicate that a tiny minority of the silica nanoparticles may be adsorbed onto the outside of the vesicles, rather than encapsulated within the vesicles. Alternatively, this may simply be a drying artefact during preparation of the TEM grid.

## Conclusions

We report the efficient one-pot synthesis of a thermoresponsive amphiphilic PDMAC_49_-P(HBA-*stat*-DAAM)_302_ diblock copolymer that can form spheres, worms or vesicles rapidly and reversibly in aqueous media at pH 7 simply by varying the solution temperature. Subsequently, an aqueous solution of this amphiphilic copolymer is cooled to 1 °C to produce spheres in the presence of glycerol-functionalized silica nanoparticles. Heating this aqueous binary mixture of organic and inorganic nanoparticles up to 50 °C induces a sphere-to-vesicle transition and concomitant *in situ* encapsulation of the silica nanoparticles. The vesicle morphology is then covalently stabilized using a disulfide-based dihydrazide to crosslink the membrane-forming P(HBA-*stat*-DAAM)_302_ chains. Importantly, the disulfide bond present within this crosslinker can be cleaved using tris(2-carboxyethyl)phosphine (TCEP), enabling the thermoresponsive behavior of the copolymer to be restored on demand. After encapsulation, excess silica nanoparticles are removed *via* multiple centrifugation cycles and the presence of silica nanoparticles within the purified vesicles is confirmed by TEM studies. Thermogravimetric analysis of the dried silica-loaded vesicles indicated a silica loading efficiency of up to approximately 86% under optimised conditions. This is significantly higher than the loading efficiencies reported for silica encapsulation during PISA,^54^ suggesting that post-polymerization encapsulation is a more effective strategy. Addition of TCEP to the silica-loaded redox-sensitive vesicles at 50 °C cleaves the disulfide bonds within the crosslinks to produce linear vesicles, with subsequent cooling to 20 °C inducing a vesicle-to-worm transition and hence release of the silica nanoparticles. This morphological transition can be monitored by SAXS studies, which confirmed that a structure factor corresponding to the encapsulated silica nanoparticles disappeared after TCEP addition followed by cooling from 50 °C to 20 °C. Postmortem DLS and TEM studies confirmed successful decrosslinking and subsequent loss of the vesicle morphology. In summary, an efficient loading strategy has been developed for redox-sensitive thermoresponsive vesicles that enables payload encapsulation to be optimized and controlled release to be conducted under mild conditions.

## Data availability

All relevant experimental data associated with this article is provided in the ESI.†

## Author contributions

S. P. A. conceived the study, obtained the funding, supervised A. C. and S. J. B. and co-wrote the paper with A. C. The crosslinkable thermoresponsive diblock copolymer was prepared by S. J. B., who also conducted initial proof-of-concept experiments. A. C. performed all the characterisation studies, analysed all the data and co-wrote the manuscript with S. P. A.

## Conflicts of interest

There are no conflicts to declare.

## Supplementary Material

SC-013-D2SC02103J-s001
