# Supplementary material for: Silica nanoparticle-loaded thermoresponsive block copolymer vesicles: a new post-polymerization encapsulation strategy and thermally triggered release
Source: Chem Sci. 2022 Aug 8;13(33):9569–79. doi: 10.1039/d2sc02103j (PMC9400661; doi:10.1039/d2sc02103j)
Supplement: SC-013-D2SC02103J-s001 [file SC-013-D2SC02103J-s001.pdf]

Electronic Supplementary Material (ESI) for Chemical Science.
This journal is © The Royal Society of Chemistry 2022

Supporting Information for:

Silica Nanoparticle-Loaded Thermoresponsive Block Copolymer Vesicles: A New Post-Polymerization Encapsulation Strategy and Thermally Triggered Release

A. Czajka, S. J. Byard and S. P. Armes*

1. Experimental
1.1 Copolymer Characterization S4

2. Supporting Analysis
2.1 Calculation of Vesicle Loading Efficiency, LE_{TGA} S7

3. Supporting Figures
3.1 ^{1}H NMR spectrum recorded for Me-DDMAT S8
3.2 ^{1}H NMR spectrum recorded for the $PDMAC_{49}$ precursor S9
3.3 DMF GPC data recorded for the $PDMAC_{49}$ precursor S10
3.4 ^{1}H NMR spectrum for $PDMAC_{49}$–P(HBA-*stat*-DAAM)$_{302}$ S11
3.5 DMF GPC trace recorded for $PDMAC_{49}$–P(HBA-*stat*-DAAM)$_{302}$ S12
3.6 DLS particle size distributions for $PDMAC_{49}$-P(HBA-*stat*-DAAM)$_{302}$ nano-objects . . S13
3.7 Fitted SAXS pattern for $PDMAC_{49}$-P(HBA-*stat*-DAAM)$_{302}$ nano-objects S14
3.8 DLS particle size distribution recorded after DS-ADH crosslinking S15
3.9 ^{1}H NMR spectra for the synthesis of the DS-ADH crosslinker S16
3.10 DLS count rate recorded during DS-ADH crosslinking S17
3.11 TEM analysis and DLS count rate after successive centrifugation cycles S18
3.12 TGA curves recorded for various copolymer concentrations S19
3.13 TGA curves recorded for various silica concentrations S20
3.14 TEM image recorded for multiple oligolamellar vesicles S21

4. Supporting Tables - S22
4.1 Loading efficiency reproducibility S22

5. SAXS Model
5.1 Vesicle model S23

References S25

1. Experimental

Materials

N,N′-Dimethyl acrylamide (DMAC, ≥ 98.5%), 2-(dodecylthiocarbonothioylthio)-2-methylpropanoic acid (DDMAT, 98%), tris(2-carboxyethyl)phosphine hydrochloride (TCEP, 98%) and 4,4′-azobis(4-cyanopentanoic acid) (ACVA, 98%) were purchased from Sigma-Aldrich (UK). 4-Dimethylaminopyridine (DMAP, ≥ 99%), *N,N'* dicyclohexylcarbodiimide (DCC, 99%), diacetone acrylamide (DAAM, 99%) and adipic acid dihydrazide ADH, ≥ 98%) were purchased from Alfa Aesar (UK). All chemicals were used as received. The Bindzil CC401 glycerol-modified aqueous silica sol (19 nm diameter; 40% w/w) was kindly supplied by Nouryon (Bohus, Sweden). Methanol was purchased from Fisher Scientific (UK) and *n*-hexane, HCl and DMF were purchased from VWR Chemicals (UK). Anhydrous dichloromethane was obtained from an in-house Grubbs solvent purification system. All solvents were HPLC-grade. HBA was donated by Scott Bader Ltd. (Wollaston, UK) and was purified by solvent extraction prior to use (see below for further details). CD_3OD was purchased from Cambridge Isotope Laboratories (UK). Deionized water was adjusted to pH 7 using NaOH and was used for all experiments.

Purification of HBA monomer via solvent extraction

HBA (100 g) was washed with *n*-hexane (20 × 100 mL) to remove diacrylate impurities. Residual solvent was removed by rotary evaporation to yield purified HBA (70 g, 70%) as a viscous colorless fluid. ^{1}H NMR (400 MHz) δ 6.37 (1H), 6.15 (1H), 5.87 (1H), 4.18 (2H), 3.59 (2H), 1.67 (4H).

Methylation of DDMAT RAFT agent

DDMAT (4.30 g, 11.8 mmol) was dissolved in anhydrous dichloromethane (30 ml) in a 100 ml round-bottomed flask, which was then cooled to 0 °C by immersion in an ice bath. DMAP (0.29 g, 2.4 mmol) and excess anhydrous methanol (2.0 g) were added to the cold stirred solution. DCC (2.72 g, 13.2 mmol) was added gradually over 5 min. This reaction mixture was allowed to warm up to 20 °C and stirred continuously for 16 h at this temperature prior to filtration to remove the insoluble dicyclohexyl urea side-product. Column chromatography was used to purify the product using dichloromethane as the eluent. This solvent was removed under vacuum to afford Me-DDMAT as an orange oil (4.21 g, 94%). ^{1}H NMR spectroscopy studies indicated a mean degree of esterification of 98%, see Figure S1.

One-Pot Synthesis of $PDMAC_{49}$-$P(HBA\text{-}stat\text{-}DAAM)_{302}$ Diblock Copolymer Vesicles

Step 1: Synthesis of PDMAC precursor by RAFT aqueous solution polymerization of DMAC

DMAC (0.680 g, 6.8 mmol), Me-DDMAT (0.060 g, 0.16 mmol, target DP = 43) and ACVA (11.1 mg, 0.040 mmol, Me-DDMAT/ACVA molar ratio = 4.0) were weighed into a 50 mL round-bottomed flask. This

solution was purged with nitrogen gas for 15 min to remove oxygen and the sealed vial was immersed in an oil bath set at 70 °C for 30 min with continuous stirring. At this point, the vial was removed from the oil bath and degassed water (2.99 g, pH 7) was added to produce a 20% w/w dispersion, which was stirred for a further 3.5 h at 70 °C. At this point, a 0.10 mL aliquot was removed for analysis by ^{1}H NMR spectroscopy and GPC. ^{1}H NMR studies indicated that more than 99% DMAC conversion was achieved within 4 h (the integrated DMAC vinyl proton signals at 5.5–7.0 ppm were compared with the integrated methyl proton signal at 2.8–3.2 ppm assigned to PDMAC), see Figure S2. DMF GPC analysis (vs. PMMA standards, see below for further details) indicated an M_p of 5 700 g mol^{-1}, see Figure S3. A mean DP of 49 was determined for the PDMAC precursor prepared during the one-pot protocol. This DP was calculated using a calibration curve of M_p vs. DP, which was constructed for a series of nine PDMAC homopolymers characterized by DMF GPC (for M_p) and end-group analysis via UV spectroscopy (for DP).[1]

Step 2: RAFT aqueous dispersion copolymerization of HBA with DAAM at pH 7 using the PDMAC$_{49}$ precursor

HBA (4.22 g, 29.2 mmol), DAAM (1.24 g, 7.3 mmol) and water (21.75 mL, pH 7) were added to a glass vial. The resulting comonomer solution was purged with nitrogen gas for 30 min. The degassed HBA/DAAM comonomer solution was added to the sealed round-bottomed flask containing the PDMAC$_{49}$ precursor, which was prepared via step 1. This sealed flask was then immersed into an oil bath set at 70 °C. A turbid free-flowing dispersion was obtained after 18 h of continuous stirring at this temperature.

^{1}H NMR spectroscopy studies indicated that more than 99% conversion was achieved for both DAAM and HBA within 18 h (as determined by comparing the DAAM/HBA vinyl proton signals at 5.5–7.0 ppm to that of the PHBA oxymethylene proton signal at 3.6 ppm and the overlapping backbone and methyl proton signals at 2.0–2.5 ppm assigned to the copolymerized HBA and DAAM repeat units, respectively), see Figure S4. DMF GPC analysis indicated an M_n of 41 600 g mol^{-1} and an M_w/M_n of 1.31, see Figure S5. An overall HBA/DAAM DP of 302 was calculated by ^{1}H NMR spectroscopy via end-group analysis using the PDMAC$_{49}$ precursor block as an 'end-group'.

Synthesis of 3,3'-dithiobis(propionohydrazide) (DS-ADH)

A literature protocol was used without modification.[2] 3,3'-Dithiodipropionic acid (5.00 g, 23.9 mmol) was dissolved in dry methanol (50 mL) containing 1-3 drops of concentrated H_2SO_4 and then refluxed under nitrogen for 1 h. The solution was concentrated under reduced pressure to less than 20 mL and then transferred to a separating funnel using diethyl ether (60 mL). The organic layer was washed with deionized water (2 × 30 mL portions) and then concentrated under reduced pressure using a rotary evaporator to yield the crude ester as an oil (light yellow colour), which was used without further purification (4.99 g, 99.8%, NMR solvent CD_3OD).

The crude ester (4.99 g) was dissolved in dry methanol (15 mL) and this solution was added dropwise into a stirred solution of hydrazine hydrate (8.00 g, 0.25 mol) in anhydrous methanol (10 mL). This reaction mixture was warmed to 35 °C and stirred overnight. TLC studies indicated complete consumption of the ester. The dihydrazide product was isolated by cooling the reaction mixture in ice for 20 min. The resulting crystals were filtered, washed with cold methanol, and dried under vacuum (0.1 mmHg, 48 h, 30 °C) to remove excess hydrazine hydrate (NMR solvent = d_6-DMSO, see Figure S9).

Silica Encapsulation

A typical protocol for silica nanoparticle encapsulation within the thermoresponsive vesicles is as follows. A 20% w/w aqueous dispersion of $PDMAC_{49}$–P(HBA-*stat*-DAAM)$_{302}$ nano-objects (2.00 g) and CC401 silica sol (2.00 g, 40% w/w in water) were added to a glass vial, which was cooled to 1 °C to produce spheres and equilibrated for 2 h. The vial was then heated to 50 °C to induce a sphere-to-vesicle transition and then equilibrated at this temperature for 2 h. The DS-ADH crosslinker (58.8 mg, 0.25 mmol; DS-ADH/DAAM molar ratio = 0.50) was added to the vial, which was immersed in an oil bath set at 50 °C and the reaction mixture was stirred for 24 h. The resulting aqueous dispersion of DS-ADH crosslinked vesicles was diluted to 1.0% w/w solids using neutral water (pH 7). Ten centrifugation-redispersion cycles (9 000 rpm for 5 min) were performed on 1.0 mL aliquots to remove excess silica nanoparticles. The purified dispersion was freeze-dried overnight and then subsequently heated in an oven at 70 °C overnight prior to thermogravimetric analysis.

Silica Release using TCEP

A typical protocol for silica release using tris(2-carboxyethyl)phosphine hydrochloride (TCEP) is as follows. 1.0 mL of a 1.0% w/w aqueous dispersion of silica-loaded $PDMAC_{49}$–P(HBA-*stat*-DAAM)$_{302}$ vesicles and TCEP (0.0035 g, TCEP/DAAM molar ratio = 1.0) were added to a glass vial, which was heated to 50 °C and left overnight. The resulting aqueous dispersion of thermoresponsive vesicles was allowed to cool to 21 °C. Variable temperature DLS studies were then performed on 0.1% w/w aqueous dispersions to determine whether decrosslinking had been achieved via disulfide bond cleavage.

1.1 Polymer Characterization

^{1}H NMR spectroscopy

^{1}H NMR spectra were recorded in CD_3OD using a 400 MHz Bruker Avance III HD 400 spectrometer with 16 scans being averaged per spectrum.

Gel permeation chromatography (GPC)

Copolymer molecular weight distributions were assessed using DMF GPC. The set-up comprised a PL guard column and two Agilent PL gel 5 µm Mixed-C columns connected in series to an Agilent 1260 Infinity GPC system equipped with both refractive index and UV-visible detectors operating at 60°C. The GPC eluent was HPLC-grade DMF containing 10 mM LiBr and the flow rate was 1.0 mL min^{-1}. Calibration was achieved using a series of ten near-monodisperse poly(methyl methacrylate) standards (ranging in M_p values from 625 to 618 000 g mol^{-1}). Chromatograms were analyzed using Agilent GPC/SEC software.

Variable Temperature Dynamic Light Scattering (DLS)

DLS studies were conducted on 0.20% w/w aqueous dispersions in disposable plastic cuvettes at the desired temperature using a Malvern Zetasizer NanoZS instrument that detects back-scattered light at an angle of 173°. Intensity-average hydrodynamic diameters were calculated via the Stokes−Einstein equation using a non-negative least squares (NNLS) algorithm. All data were averaged over three consecutive runs. Where appropriate, intensity-average hydrodynamic diameters were converted into volume-average hydrodynamic diameters using Malvern Zetasizer Software (Version 7.01).

UV−Visible Absorption Spectroscopy

UV−visible absorption spectra were recorded between 200 and 800 nm using a PC-controlled UV-1800 spectrophotometer at 25°C. A 1.0 cm path length quartz cell was used and mean DPs were calculated for a series of nine PDMAC homopolymers by end-group analysis. Here the molar extinction coefficient of 16300 ± 160 mol^{-1} dm^3 cm^{-1} previously determined for the DDMAT RAFT agent was assumed to be valid for the trithiocarbonate-capped PDMAC chains.[1]

Transmission Electron Microscopy (TEM)

Copper/palladium TEM grids (Agar Scientific, UK) were surface-coated in-house to yield a thin film of amorphous carbon. The grids were then plasma glow-discharged for 30 s to create a hydrophilic surface. Individual samples (0.20% w/w, 5.0 µL) were adsorbed onto the freshly glow-discharged grids for 1 min and then blotted with filter paper to remove excess solution. When silica was not present in the sample, it was necessary to stain the aggregates using a uranyl formate solution (0.75% w/v, 5.0 µL), which was placed on the sample-loaded grid for 20 s, and then carefully blotted to remove excess stain. The grids were then dried using a vacuum hose. Imaging was performed on a Technai T12 Spirit instrument at 120 kV equipped with a Gatan 1 k CCD camera.

Rheology

An AR-G2 rheometer equipped with a variable-temperature Peltier plate and a 40 ml 2° aluminum cone was used for all experiments. Temperature sweeps were conducted using a constant strain of 1.0% and a fixed angular frequency of 1.0 rad s^{-1}. Prior to the temperature sweep, the aqueous copolymer dispersion was equilibrated at 1 °C for 10 min. A ramp rate of 1 °C min^{-1} was used for all experiments.

Thermogravimetric Analysis (TGA)

Thermogravimetric analyses were conducted using a Perkin-Elmer Pyris 1 TGA instrument. Excess non-adsorbed silica nanoparticles remaining after the silica encapsulation process were removed via ten centrifugation-redispersion cycles (9 000 rpm for 5 min), with each successive supernatant being carefully decanted and replaced with deionized water. TEM studies confirmed removal of the excess silica nanoparticles using this purification protocol (see Figure S11). Purified copolymer dispersions were freeze-dried overnight and subsequently dried in an oven at 70 °C for 24 h to remove water. The resulting dried silica-loaded vesicles were heated in air up to 800 °C at 10 °C min^{-1}. The observed mass loss was attributed to pyrolysis of the copolymer component, with the remaining white incombustible residues being assumed to be that of pure silica (SiO_2). The silica loading efficiency, LE_{TGA}, was calculated from the residual mass, see Section 2.3.

Small-angle X-ray Scattering (SAXS)

SAXS patterns were recorded at a synchrotron facility (station I22 at Diamond Light Source, Didcot, Oxfordshire, UK). A monochromatic X-ray beam (λ = 0.124 nm) and a 2D Pilatus 2M pixel detector (Dectris, Baden, Switzerland), were used for these experiments. A q range of 0.02−2.00 nm^{-1} was used for measurements, where $q = (4\pi \sin \theta)/\lambda$, corresponds to the modulus of the scattering vector and θ is half of the scattering angle. Measurements were recorded using a flow-through cell which contains a glass capillary with a mean diameter of 1.8 mm. X-ray scattering data were reduced (integrated, normalized, and background subtracted) using DAWN and SAXS Utilities software. The scattering intensity of water was used for absolute scale calibration of the X-ray scattering patterns. Irena SAS macros for Igor Pro were used for modeling and further SAXS analysis.

2. Supporting Analysis

2.1 Calculation of Vesicle Loading Efficiency, *LE*$_{TGA}$

The dried silica nanoparticles used in this study lose approximately 3.8% mass on heating to 800 °C, see Figure 5. This is attributed to the combination of surface dehydration and pyrolysis of the surface glycerol groups. When calculating the silica content of the vesicles, this mass loss must be taken into account using equation **S1**:

$$RM_c = \frac{RM_{Sample}}{(RM_{Si}/100)} \quad \textbf{S1}$$

Here RM_c corresponds to the corrected residual mass, RM_{Sample} corresponds to the residual mass for a particular sample and RM_{Si} corresponds to the residual mass observed for silica nanoparticles alone (i.e. 100 – 3.8 = 96.2%). For example, the first entry in Table 1 (5% w/w copolymer, 20% w/w silica) produced RM_{Sample} = 20.3% (see TGA Figure S12). Thus it follows that:

$$RM_c = \frac{20.3}{(96.2/100)} = 21.1\%$$

Once RM_c has been determined for a particular 'copolymer plus silica' formulation, the vesicle loading efficiency determined by TGA, *LE*$_{TGA}$, can be calculated using equation **S2**:

$$LE_{TGA} = 100\left[\frac{10\left(\frac{RM_c}{1 - RM_c}\right)}{[silica]_0}\right] = X\ \% \quad \textbf{S2}$$

For the 5% w/w copolymer plus 20% w/w silica sample shown in Table 1, we calculate:

$$LE_{TGA} = 100\left[\frac{10\left(\frac{0.211}{1-0.211}\right)}{20}\right] = 13.4\%$$

3. Supporting Figures

3.1 ^{1}H NMR spectrum recorded for Me-DDMAT

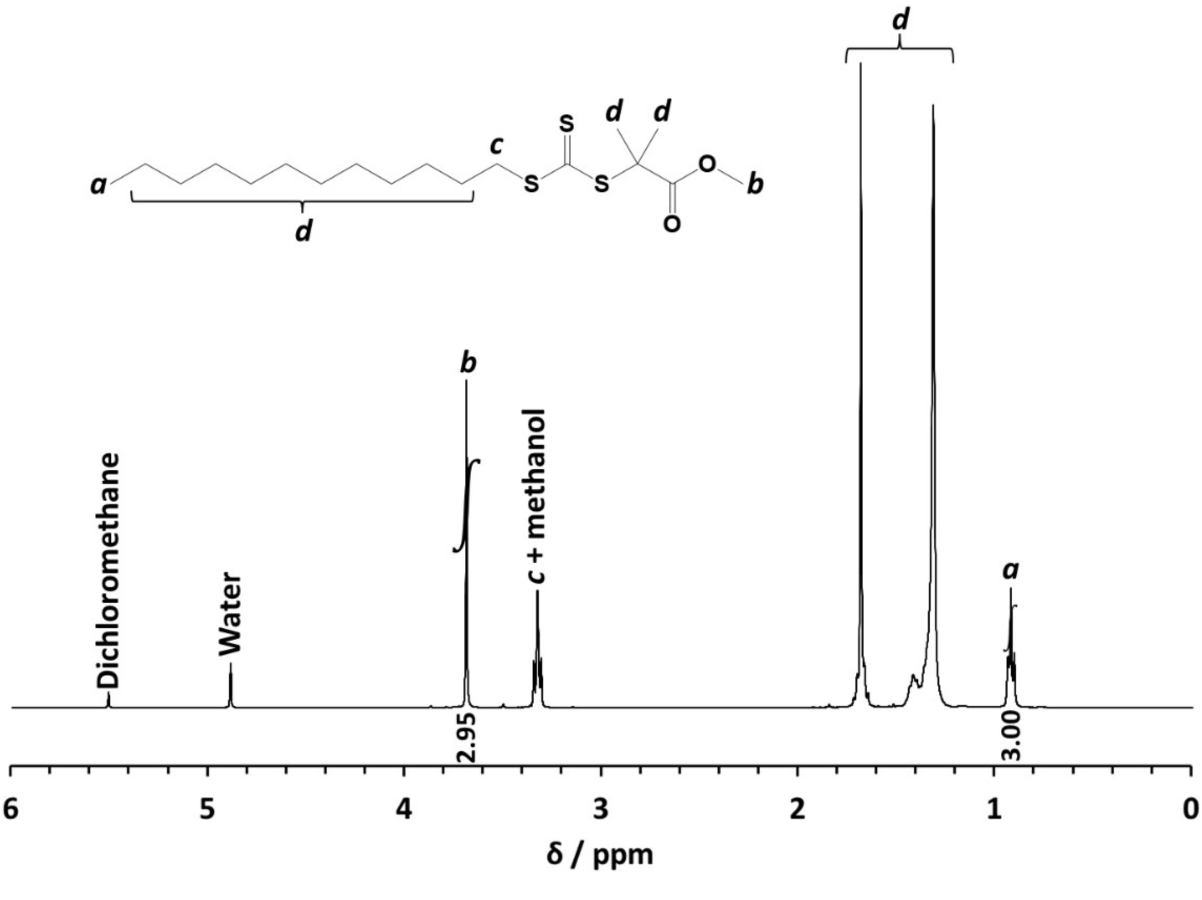

Figure S1. ^{1}H NMR spectrum recorded in CD_3OD for Me-DDMAT after purification by column chromatography. Signals *a* and *b* were used to calculate a mean degree of esterification of 98%.

3.2 ^{1}H NMR spectrum recorded for the PDMAC$_{49}$ precursor

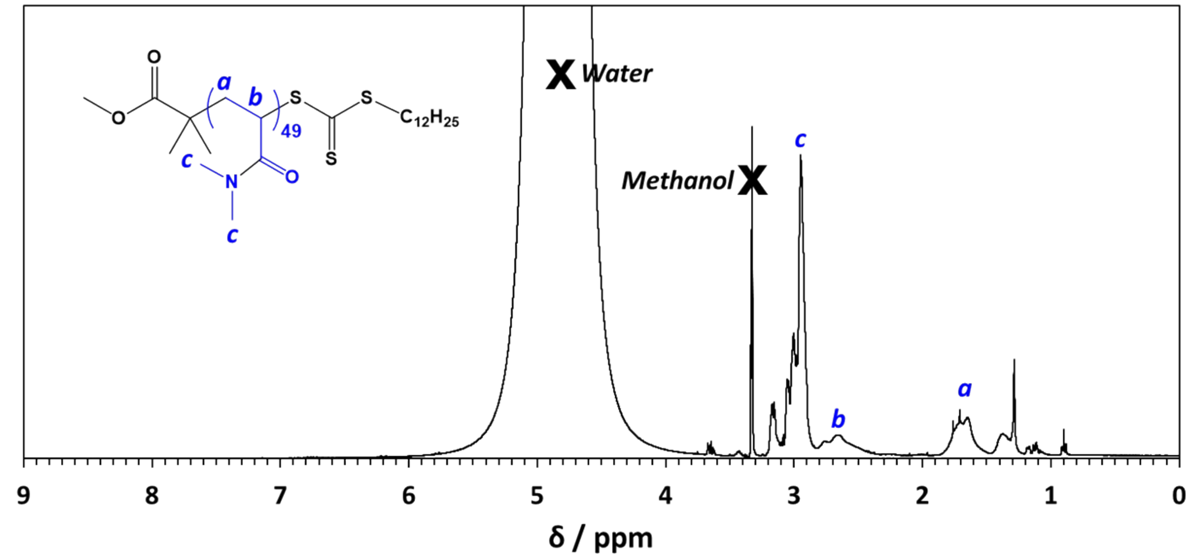

Figure S2. Partially assigned ^{1}H NMR spectrum recorded in CD_3OD for the PDMAC$_{49}$ precursor prepared at pH 7.

3.3 DMF GPC data recorded for the $PDMAC_{49}$ precursor

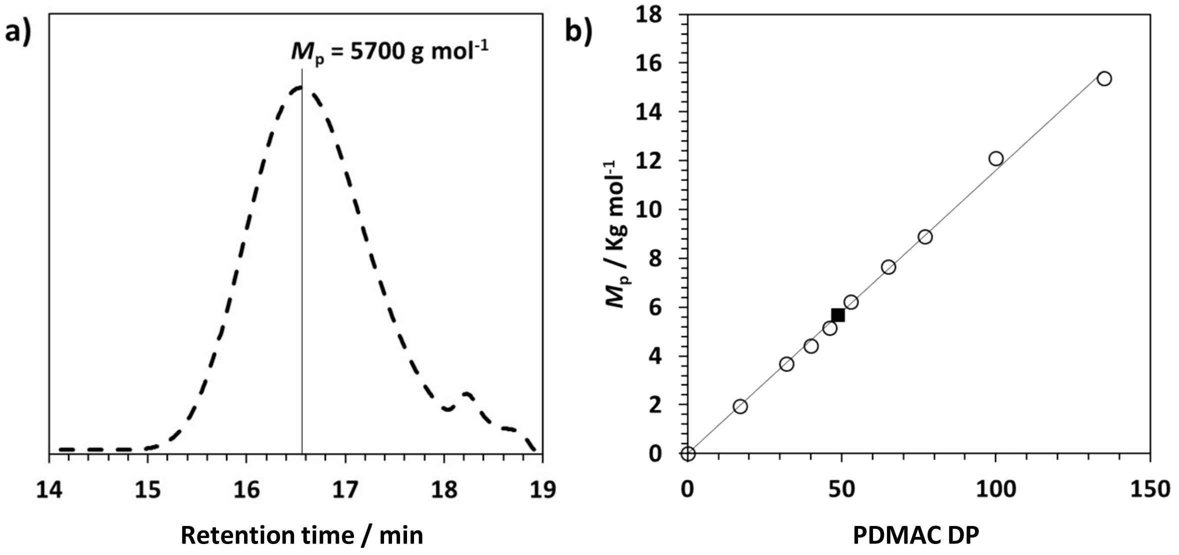

Figure S3. (a) DMF GPC trace recorded for the $PDMAC_{49}$ precursor prepared using the one-pot aqueous PISA protocol outlined in Scheme 1. (b) Calibration plot to determine the mean DP of the PDMAC precursor (see black square) obtained according to Scheme 1. Linear relationship between M_p (determined by DMF GPC analysis using a refractive index detector) and mean PDMAC DP (determined by UV spectroscopy using the Beer-Lambert calibration plot constructed for DDMAT, see ref. 1) for a series of PDMAC homopolymers prepared by RAFT solution polymerization of DMAC in 1,4-dioxane using DDMAT and purified by precipitation (open circles).

3.4 ^{1}H NMR spectrum for PDMAC$_{49}$–P(HBA-*stat*-DAAM)$_{302}$

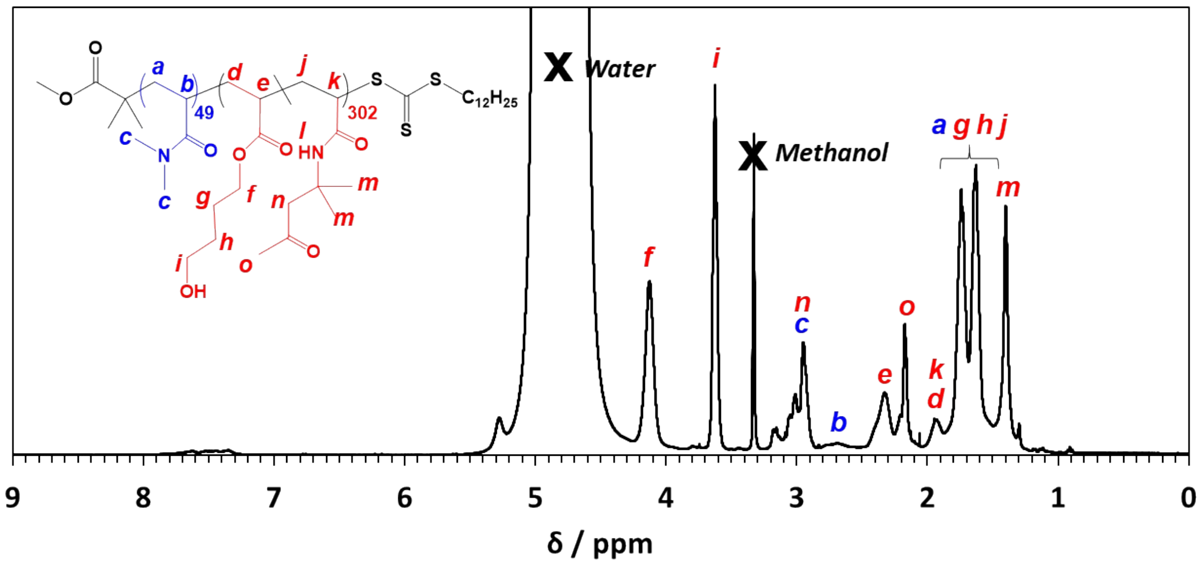

Figure S4. Assigned ^{1}H NMR spectrum (CD_3OD) recorded for the PDMAC$_{49}$–P(HBA-*stat*-DAAM)$_{302}$ diblock copolymer prepared at pH 7 according to the one-pot protocol shown in Scheme 1.

3.5 DMF GPC trace recorded for PDMAC$_{49}$−P(HBA-*stat*-DAAM)$_{302}$

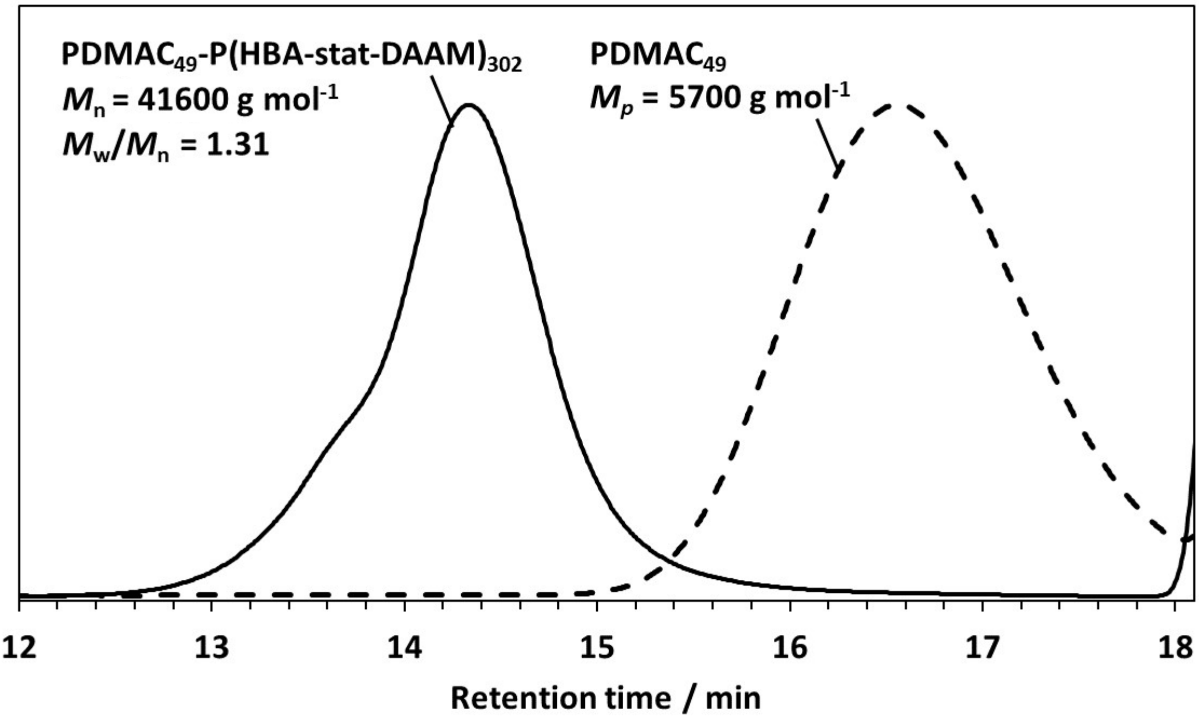

Figure S5. DMF GPC traces recorded for the PDMAC$_{49}$ precursor (dashed trace) and the PDMAC$_{49}$-P(HBA-*stat*-DAAM)$_{302}$ diblock copolymer (solid trace) prepared at pH 7 according to the one-pot protocol shown in Scheme 1.

3.6 DLS particle size distributions for PDMAC$_{49}$-P(HBA-*stat*-DAAM)$_{302}$ nano-objects

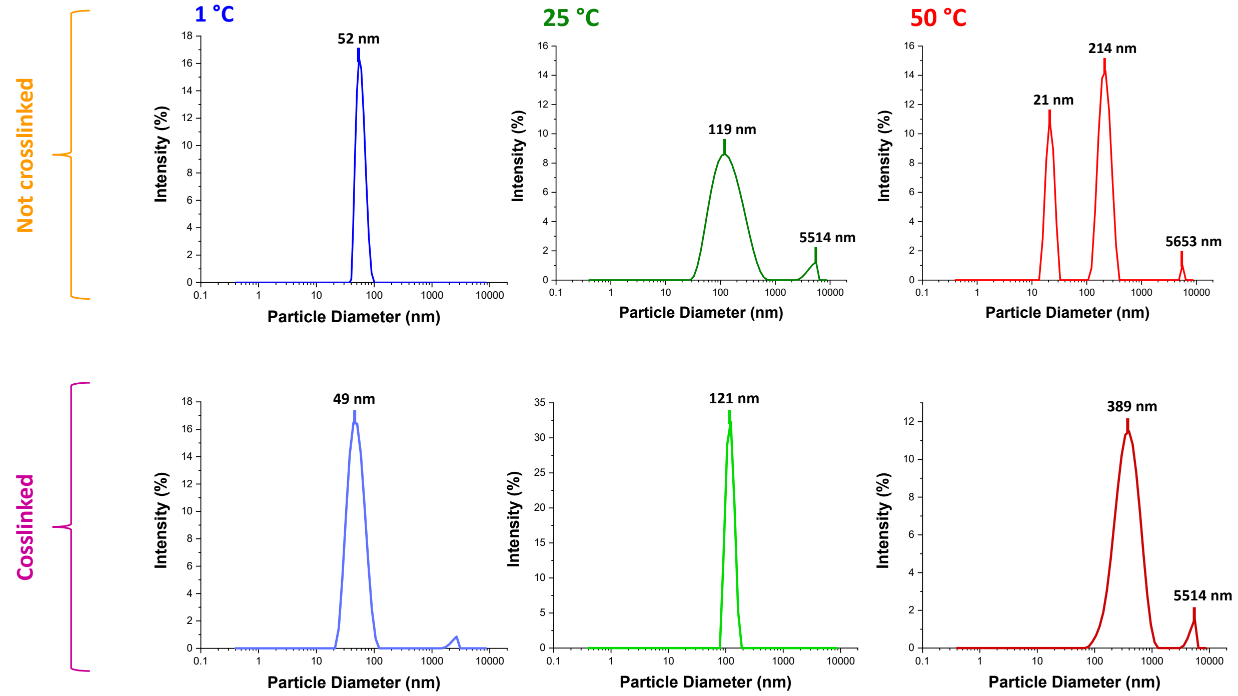

Figure S6. Intensity-average particle size distributions obtained by DLS studies of PDMAC$_{49}$-P(HBA-*stat*-DAAM)$_{302}$ nano-objects at 0.10%w/w, both prior to and after crosslinking at 2, 25 or 50 °C. The respective temperature for each DLS experiment is indicated for each panel.

3.7 Fitted SAXS pattern for PDMAC$_{49}$-P(HBA-*stat*-DAAM)$_{302}$ nano-objects

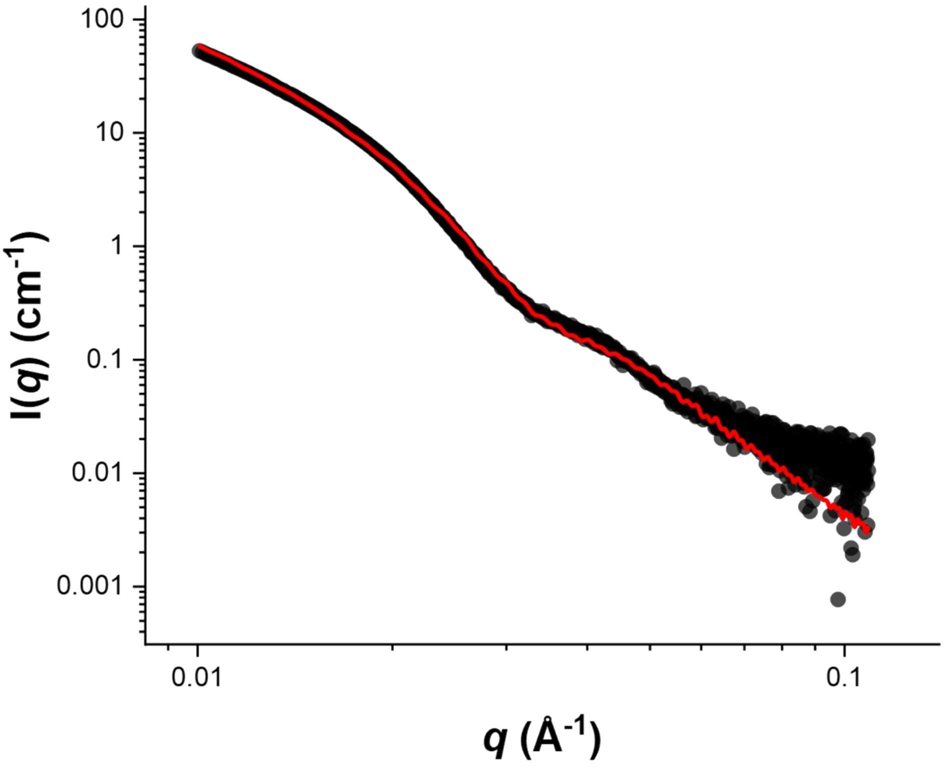

Figure S7. Small-angle X-ray scattering (SAXS) pattern recorded for PDMAC$_{49}$-P(HBA-*stat*-DAAM)$_{302}$ nano-objects at 1.0% w/w and 50 °C. Red line corresponds to the data fit obtained using a sophisticated vesicle model, see Section 5 in SI.

3.8 DLS particle size distribution recorded after DS-ADH crosslinking

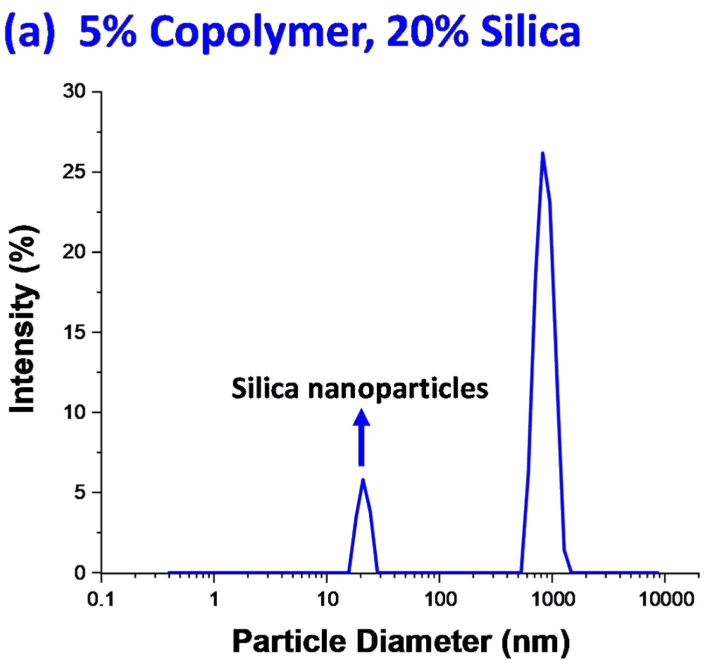

Figure S8. Intensity-average particle size distributions obtained after crosslinking with DS-ADH prior to removal of excess silica nanoparticles. The two populations correspond to the excess silica nanoparticles and the DS-ADH crosslinked vesicles. Initial copolymer and silica concentrations were 5% w/w and 20% w/w, respectively.

3.9 ^{1}H NMR spectra for the synthesis of the DS-ADH crosslinker

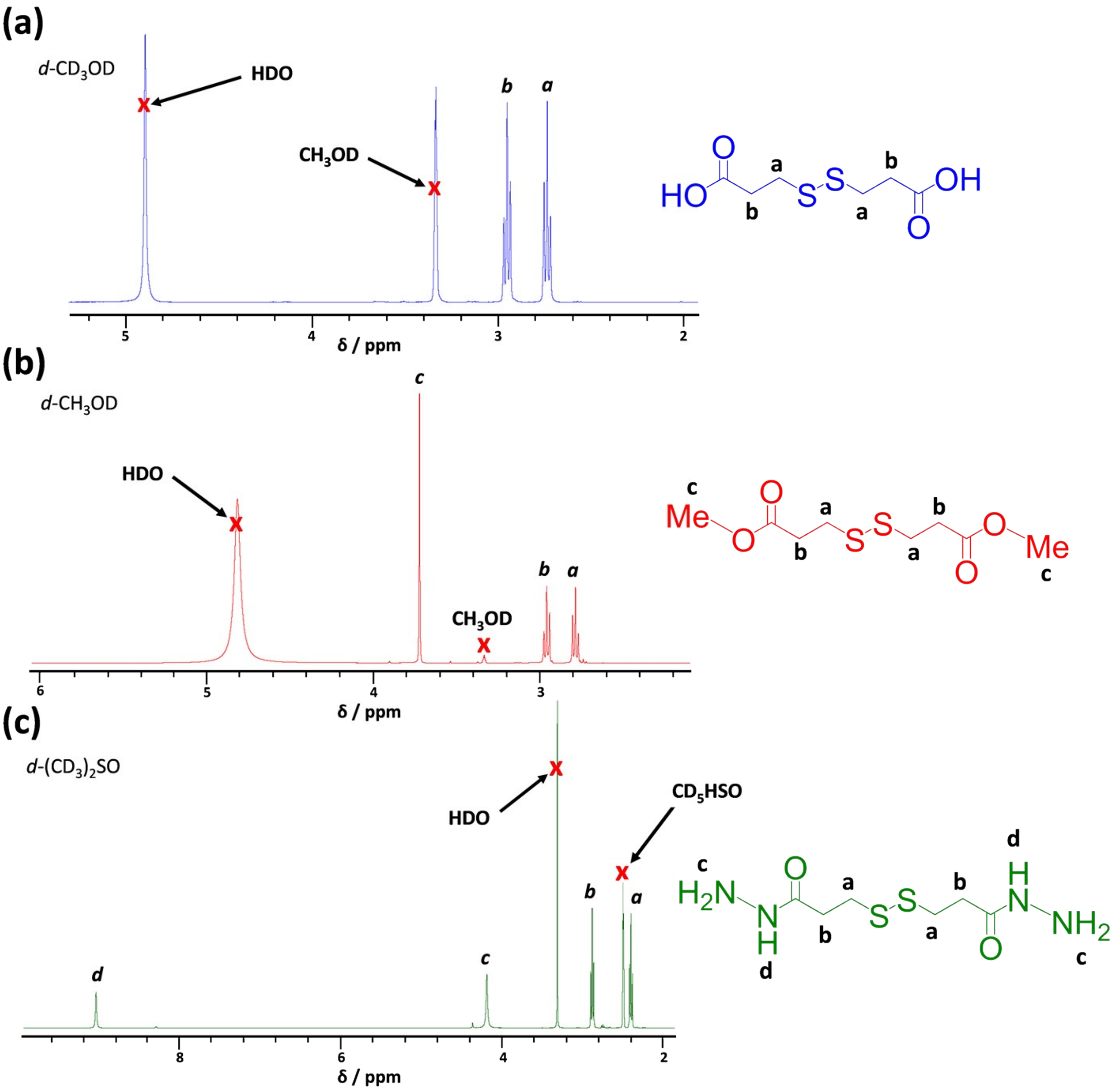

Figure S9. ^{1}H NMR spectra recorded for the synthesis of the disulfide-based crosslinker DS-ADH: (a) the dicarboxylic acid precursor, (b) the crude diester obtained after esterification and (c) the final DS-ADH product. The corresponding deuterated solvents (d_4-methanol, d_6-DMSO) and peak labels are shown.

3.10 DLS count rate recorded during DS-ADH crosslinking

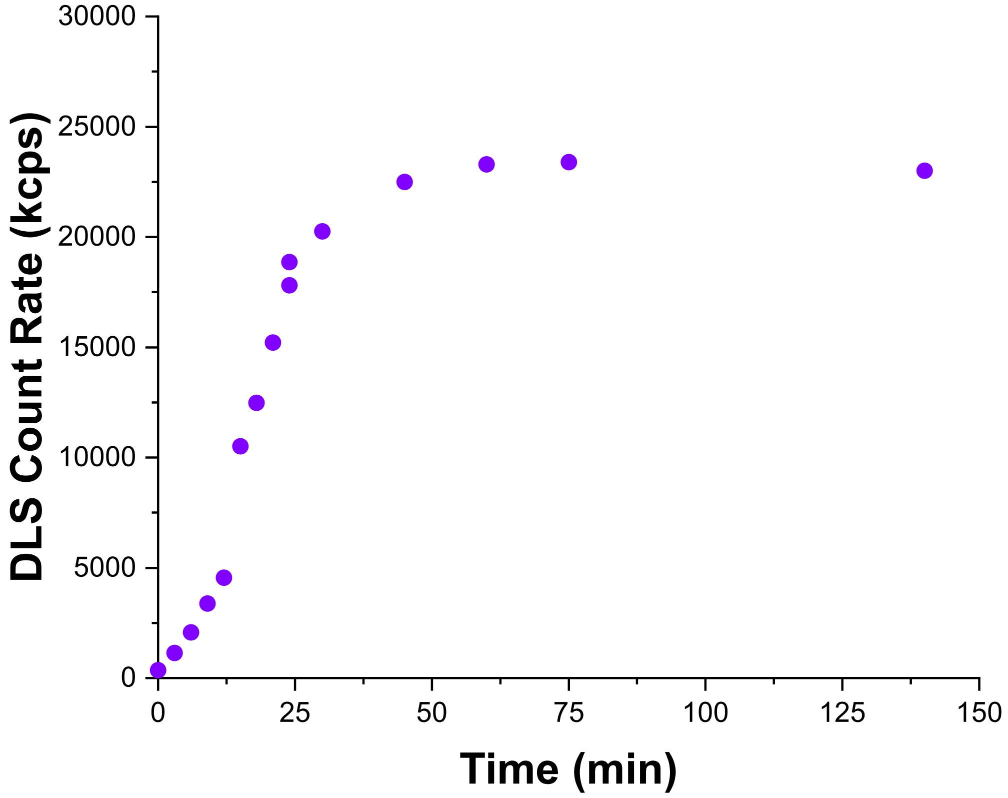

Figure S10. Derived count rate determined for aliquots periodically removed during DS-ADH crosslinking of vesicles at 50 °C. Conditions: [copolymer]$_0$ = 10% w/w and [silica]$_0$ = 5% w/w.

3.11 TEM analysis and DLS count rate after successive centrifugation cycles

(a)

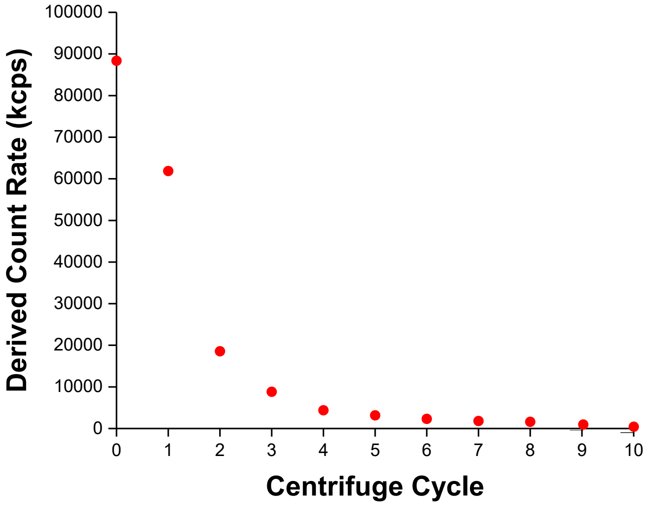

(b)

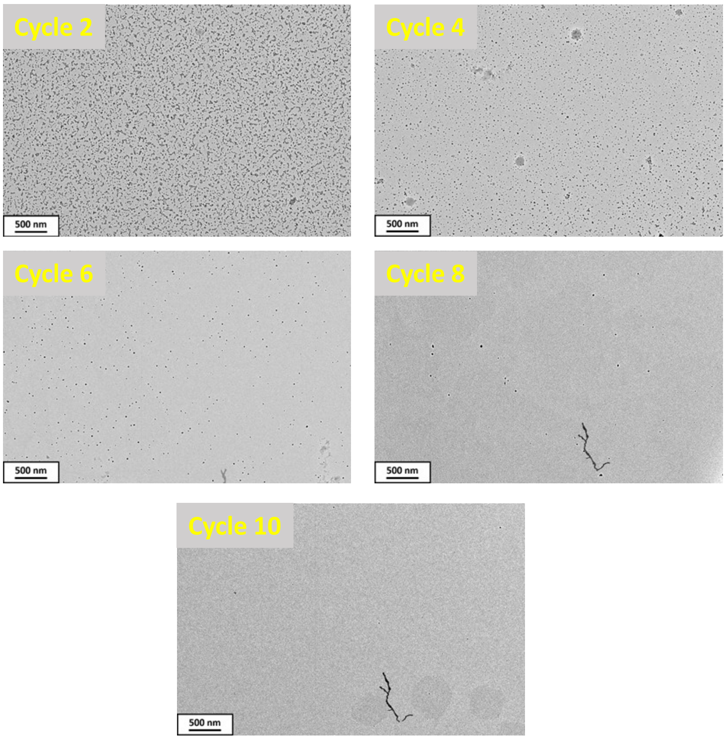

Figure S11. (a) DLS derived count rate (or scattered light intensity) determined for successive aqueous supernatants obtained after ten centrifugation-redispersion cycles to remove excess silica nanoparticles from DS-ADH crosslinked PDMAC$_{49}$-P(HBA-*stat*-DAAM)$_{302}$ vesicles prepared at [copolymer]$_0$ = 5% w/w in the presence of [silica]$_0$ = 20% w/w [N.B. The copolymer dispersion was diluted to 1.0% w/w prior to centrifugation]. (b) Corresponding TEM images recorded for selected dried supernatants during this centrifugal purification protocol.

3.12 TGA curves recorded for various copolymer concentrations

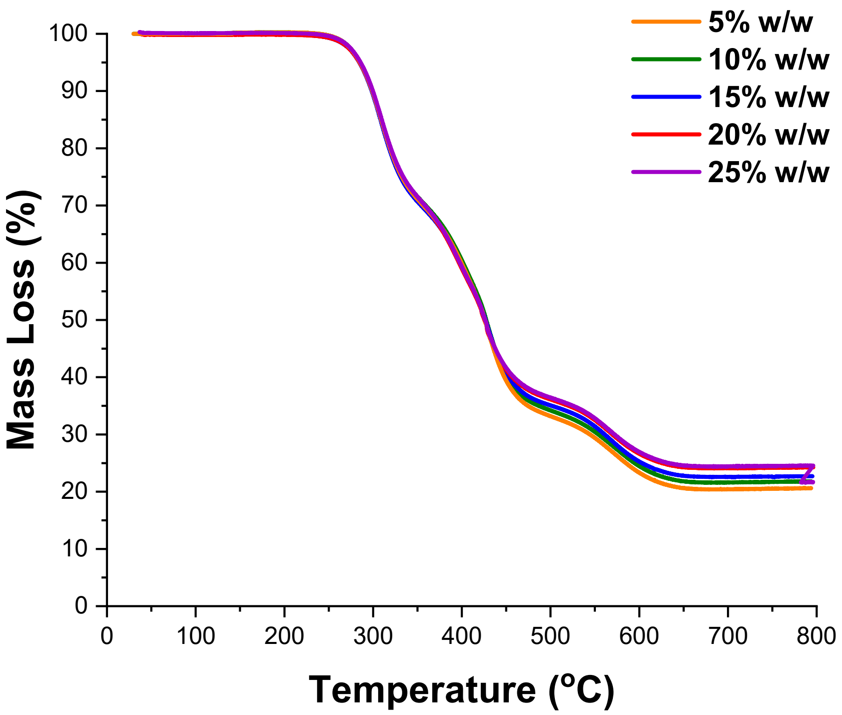

Figure S12. Thermogravimetric analysis curves recorded for a series of DS-ADH crosslinked PDMAC$_{49}$-P(HBA-*stat*-DAAM)$_{302}$ vesicles prepared at [silica]$_0$ = 20% w/w with varying [copolymer]$_0$ (5-25% w/w, see Figure) after purification via ten centrifugation-redispersion cycles. Each sample was freeze-dried for 12 h and then dried in an oven at 75 °C for 12 h prior to thermogravimetric analysis.

3.13 TGA curves recorded for various silica concentrations

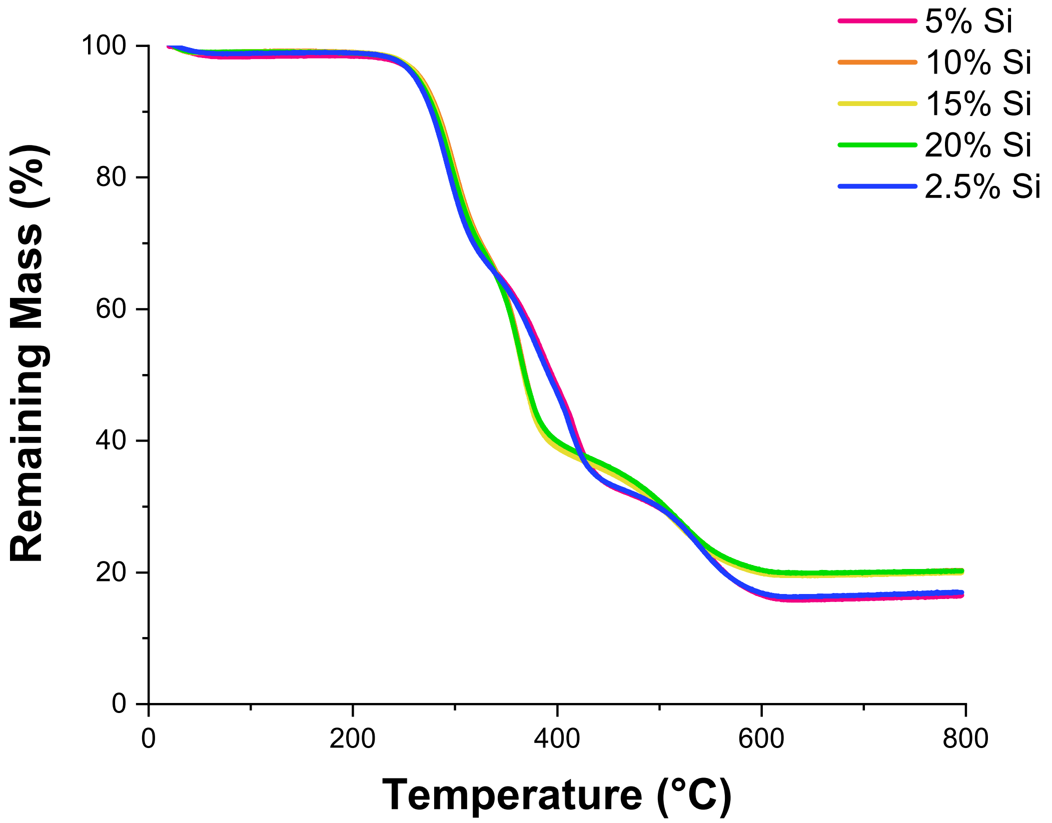

Figure S13. Thermogravimetric analysis curves recorded for a series of DS-ADH crosslinked PDMAC$_{49}$-P(HBA-*stat*-DAAM)$_{302}$ vesicles prepared at a constant copolymer concentration of 10% w/w while varying the [silica]$_0$ from 5% w/w to 20% w/w. The resulting silica-loaded vesicles were purified via ten centrifugation-redispersion cycles to remove excess silica nanoparticles, freeze-dried for 12 h and then dried in an oven at 75 °C for 12 h prior to thermogravimetric analysis.

3.14 TEM image recorded for multiple oligolamellar vesicles

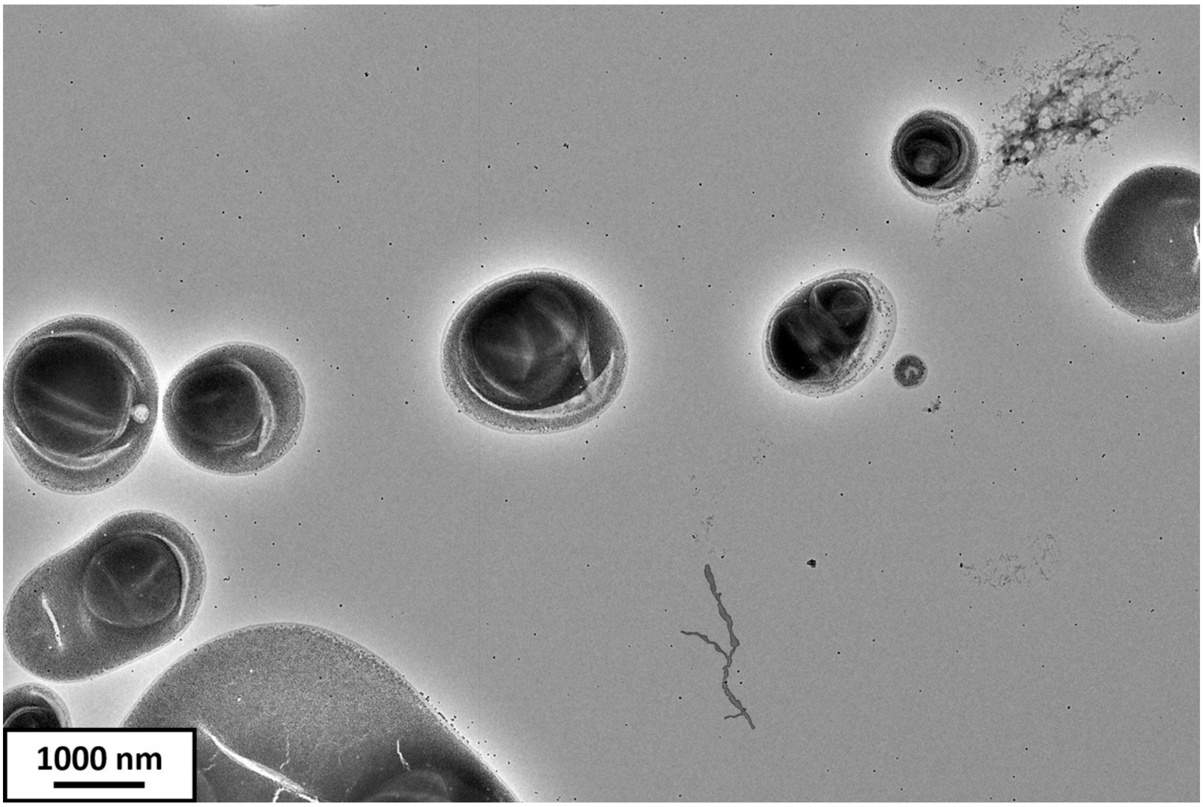

Figure S14. DS-ADH crosslinked PDMAC$_{49}$-P(HBA-*stat*-DAAM)$_{302}$ vesicles loaded with silica nanoparticles prepared at a copolymer concentration of 20% w/w in the presence of a 20% w/w aqueous dispersion of silica nanoparticles. Excess silica removed via ten centrifugation-redispersion cycles. Close inspection reveals the presence of multiple oligolamellar vesicles (i.e. vesicles within vesicles).

4. Supporting Tables

4.1 Loading Efficiency Reproducibility

Table S1. The loading efficiency determined from TGA (LE_{TGA}) for three repeat runs at $[copolymer]_0$ = 10% w/w and $[silica]_0$ = 2.5% w/w with DS-ADH crosslinking conducted at either 50 or 37 °C. The data obtained for each formulation shown in Table 1 is also included.

	Z-Average (nm)	TGA silica content (%)	LE_{TGA}
P10 Si2.5 (50 °C)	1045 (0.28)	16.9	85.6
Repeat 1	1020 (0.19)	16.3	81.6
Repeat 2	1132 (0.25)	16.2	81.0
Repeat 3	1231 (0.35)	17.5	88.4
P10 Si2.5 (37 °C)	860 (0.34)	13.3	63.6
Repeat 1	920 (0.38)	13.0	62.5
Repeat 2	1020 (0.34)	13.6	65.9
Repeat 3	969 (0.25)	14.0	67.0

5. SAXS Model

In general, the intensity of X-rays scattered by a dispersion of nano-objects [usually represented by the scattering cross-section per unit sample volume, $\frac{d\Sigma}{d\Omega}(q)$] can be expressed as:

$$\frac{d\Sigma}{d\Omega}(q) = NS(q)\int_0^\infty \dots \int_0^\infty F(q,r_1,\dots,r_k)^2\Psi(r_1,\dots,r_k)dr_1,\dots,dr_k \qquad \textbf{S3}$$

where $F(q,r_1,\dots,r_k)$ is the form factor, $r_1,\dots,r_k$ is a set of k parameters describing the structural morphology, $\Psi(r_1,\dots,r_k)$ is the distribution function, $S(q)$ is the structure factor and N is the nano-object number density per unit volume expressed as:

$$N = \frac{\varphi}{\int_0^\infty \dots \int_0^\infty V(r_1,\dots,r_k)\Psi(r_1,\dots,r_k)dr_1,\dots,dr_k} \qquad \textbf{S4}$$

where $V(r_1,\dots,r_k)$ is the volume of the nano-object and φ is the volume fraction of nano-objects.

5.1 Vesicle model

The vesicle form factor in Equation S3 is expressed as:

$$F_{v_{mic}}(q) = N_v^2\beta_s^2A_m^2(q) + N_v\beta_c^2F_c(q,R_g) + N_v(N_v-1)\beta_c^2A_{vc}^2(q) + 2N_v^2\beta_c\beta_sA_m(q)A_{vc}(q) \qquad \textbf{S5}$$

where R_g is the radius of gyration of the PGMA corona block. The X-ray scattering length contrast for the core block and the corona block is given by $\beta_s = V_s(\xi_s - \xi_{sol})$ and $\beta_c = V_c(\xi_c - \xi_{sol})$, respectively. Here ξ_s, ξ_c and ξ_{sol} are the X-ray scattering length densities of the core block (ξ_{PDMAC} = 9.35 × 10^{10} cm^{-2}), the corona block ($\xi_{PHBA-stat-PDAAM}$ = 9.20 × 10^{10} cm^{-2}) and the solvent (ξ_{sol} = 9.42 × 10^{10} cm^{-2}), respectively. V_s and V_c are the volumes of

the core block ($V_{PDMAC49}$) and the corona block ($V_{PHBA240-stat-PDAAM60}$), respectively. These volumes were calculated using Equation S6:

$$V_{block} = \frac{MW\ x\ DP}{\rho\ x\ N_a} \qquad \textbf{S6}$$

where MW corresponds to the monomer molecular weight, DP is the mean degree of polymerization of the block, ρ is the density and N_a is Avogadro's constant.

The amplitude of the membrane self-term is:

$$A_m(q) = \frac{V_{out}\varphi(qR_{out}) - V_{in}\varphi(qR_{in})}{V_{out} - V_{in}} exp\left(-\frac{q^2{\sigma_{in}}^2}{2}\right) \qquad \textbf{S7}$$

where $R_{in} = R_m - \frac{1}{2}T_m$ is the inner radius of the membrane and $R_{out} = R_m + \frac{1}{2}T_m$ is the outer radius of the membrane (R_m is the radius from the centre of the vesicle to the centre of the membrane), and $V_{in} = \frac{4}{3}\pi {R_{in}}^3$ and $V_{out} = \frac{4}{3}\pi {R_{out}}^3$. The exponent term in Equation S7 represents a sigmoidal interface between the two blocks, with a width σ_{in} accounting for a decaying scattering length density at the membrane surface. The value of σ_{in} was fixed at 2.5 during fitting. The amplitude of the vesicle corona self-term is expressed as:

$$A_{vc}(q) = \Psi(qR_g)\frac{1}{2}\left[\frac{sin[q(R_{out} + R_g)]}{q(R_{out} + R_g)} + \frac{sin[q(R_{in} - R_g)]}{q(R_{in} - R_g)}\right] \qquad \textbf{S8}$$

where the term outside the square brackets is the form factor amplitude of the corona block such that

$$\Psi(qR_g) = \frac{1 - exp(-qR_g)}{(qR_g)^2} \qquad \textbf{S9}$$

For the vesicle model, it is assumed that two parameters are polydisperse: the radius from the centre of the vesicle to the centre of the membrane (R_m) and the membrane thickness (T_m). Each parameter is considered to have a Gaussian distribution, hence the polydispersity function in Equation S3 can be expressed in each case as:

$$\Psi(r_1 r_2) = \frac{1}{\sqrt{2\pi\sigma_{R_s}^{\ 2}}}\exp\left(-\frac{(r_1 - R_m)^2}{2\sigma_{R_m}^{\ 2}}\right)\frac{1}{\sqrt{2\pi\sigma_{T_m}^{\ 2}}}\exp\left(-\frac{(r_1 - T_m)^2}{2\sigma_{T_m}^{\ 2}}\right) \quad \textbf{S10}$$

Where σ_{R_m} and σ_{T_m} are the standard deviations for R_m and T_m, respectively. According to Equation S4, the number density per unit volume for the vesicle model can be expressed as:

$$N = \frac{\varphi}{\int_0^\infty \int_0^\infty V(r_1,r_2)\Psi(r_1,r_2)dr_1 dr_2} \quad \textbf{S11}$$

Here φ is the copolymer volume fraction and $V(r_1,r_2)$ is the total copolymer volume $[V(r_1,r_2) = (V_s + V_c)N_v(r_1,r_2)]$.

Programming tools within the Irena SAS Igor Pro macros were used to implement this vesicle scattering model.[3]

References

1 S. J. Byard, M. Williams, B. E. McKenzie, A. Blanazs and S. P. Armes, *Macromolecules*, 2017, **50**, 1482–1493.

2 K. P. Vercruysse, D. M. Marecak, J. F. Marecek and G. D. Prestwich, *Bioconjugate Chem.*, 1997, **8**, 686–694.

3 J. Ilavsky and P. R. Jemian, *J. Appl. Crystallogr.*, 2009, **42**, 347–353.
